# Expression of varied GFPs in *Saccharomyces cerevisiae*: codon optimization yields stronger than expected expression and fluorescence intensity

**DOI:** 10.1038/srep35932

**Published:** 2016-10-26

**Authors:** Misato Kaishima, Jun Ishii, Toshihide Matsuno, Nobuo Fukuda, Akihiko Kondo

**Affiliations:** 1 Department of Chemical Science and Engineering, Graduate School of Engineering, Kobe University, 1-1 Rokkodai, Nada, Kobe 657-8501, Japan; 2 Graduate School of Science, Technology and Innovation, Kobe University, 1-1 Rokkodai, Nada, Kobe 657-8501, Japan; 3 Biomedical Research Institute, National Institute of Advanced Industrial Science and Technology (AIST), Higashi, Tsukuba, Japan

## Abstract

Green fluorescent protein (GFP), which was originally isolated from jellyfish, is a widely used tool in biological research, and homologs from other organisms are available. However, researchers must determine which GFP is the most suitable for a specific host. Here, we expressed GFPs from several sources in codon-optimized and non-codon-optimized forms in the yeast *Saccharomyces cerevisiae*, which represents an ideal eukaryotic model. Surprisingly, codon-optimized mWasabi and mNeonGreen, which are typically the brightest GFPs, emitted less green fluorescence than did the other five codon-optimized GFPs tested in *S. cerevisiae*. Further, commercially available GFPs that have been optimized for mammalian codon usage (e.g., EGFP, AcGFP1 and TagGFP2) unexpectedly exhibited extremely low expression levels in *S. cerevisiae*. In contrast, codon-optimization of the GFPs for *S. cerevisiae* markedly increased their expression levels, and the fluorescence intensity of the cells increased by a maximum of 101-fold. Among the tested GFPs, the codon-optimized monomeric mUkG1 from soft coral showed the highest levels of both expression and fluorescence. Finally, the expression of this protein as a fusion-tagged protein successfully improved the reporting system’s ability to sense signal transduction and protein–protein interactions in *S. cerevisiae* and increased the detection rates of target cells using flow cytometry.

Green fluorescent protein (GFP) was discovered more than 50 years ago^1^ and has fueled a new era in cell and molecular biology. GFP comprises 238 amino acid residues (approximately 27 kDa) with a β-barrel structure consisting of eleven β-strands, and it emits green fluorescence when exposed to blue light^2^. GFP requires no external cofactors other than oxygen to form the chromophore, unlike other proteins that require cofactors or substrates for their activities (e.g., opsin, β-glucuronidase, β-galactosidase, chloramphenicol acetyl-transferase, and luciferase)^3^. Therefore, GFP represents an excellent reporter gene and fusion tag for the analysis of gene regulation, protein localization, and specific organelle labeling in almost every organism^4^,^5^,^6^,^7^,^8^.

The budding yeast *Saccharomyces cerevisiae* is an ideal eukaryotic model organism for studying protein localization due to the ease and tractability of its genetic modification^7^. Recent studies have applied this tool widely, for example in genome-wide approaches to analyze messenger RNA (mRNA) abundance^9^,^10^, transcriptional regulation^11^, protein abundance or localization^3^,^12^,^13^, and protein–protein interactions^14^,^15^. In many cases, these studies used GFP or other chromophoric proteins for the facile tracking and monitoring of target proteins. Further, the use of flow cytometry has accelerated the screening of target cells. GFP has been widely applied to the yeast *S. cerevisiae*, thus rendering it a useful tool for a wide variety of biological studies.

Several varieties of GFPs were tested in this study, and their properties are listed in Table 1. The GFP that was first isolated from jellyfish *Aequorea victoria* forms a weakly associated dimer that exhibits a low molar extinction coefficient (ε) and two excitation maxima (395 nm and 475 nm)^16^,^17^,^18^. Enhanced GFP (EGFP), a mutant of the original GFP that was developed through mutagenesis studies, is among the most popular and widely used monomeric GFPs, and it exhibits a single excitation peak at 488 nm^19^ (Table 1). EGFP contains codons that were optimized for mammalian cell expression and S65T and F64L mutations that improve the spectral characteristics, fluorescence intensity and stability of the protein and increased protein maturation efficiency at 37 °C^19^,^20^.

Recently, GFP variants have been discovered in a wide range of sources, including various *Aequorea* species^21^,^22^, copepods^23^, amphioxus^24^,^25^ and reef corals^26^,^27^,^28^,^29^, and these variants are now commercially available. For example, AcGFP1, a brilliant green monomeric GFP variant isolated from *Aequorea coerulescens*, exhibits 94% sequence homology to EGFP^21^ (Table 1). The monomeric and pH-stable GFP, TagGFP2 (mTagGFP), which is the improved variant of TagGFP derived from *Aequorea macrodactyla*, exhibits 80% sequence homology to EGFP^22^ (Table 1). The coral-derived GFPs mUkG1^26^, ZsGreen^27^ and mWasabi^29^, bear surprisingly low sequence similarity to the GFPs that were originally identified in *Aequorea* species. Monomeric mUkG1 emits intense fluorescence and is extremely stable against high pH, owing to the introduction of five mutations to the dimeric wild-type fluorescent protein UkG1, which was isolated from the soft coral *Sarcophyton* sp.^26^ (Table 1). Tetrameric ZsGreen is a *Zoanthus* sp. fluorescent protein variant with a single amino acid substitution that enhances the emission characteristics; the protein has a substantially higher quantum yield (QY)^27^ (Table 1). The bright, monomeric green fluorescent protein mWasabi is a variant of monomeric mTFP1, which is derived from the tetrameric cyan fluorescent protein cFP484 of *Clavularia* sp.^29^ (Table 1). mNeonGreen is a monomeric yellow-green fluorescent protein that emits the brightest green or yellow fluorescence reported to date; this protein was derived from the tetrameric yellow fluorescent protein (LanYFP) of the cephalochordate *Branchiostoma lanceolatum* by structure-guided directed evolution^24^ (Table 1).

Thus, a wide range of GFPs are now available. These proteins generating green fluorescence are crucially important as versatile experimental tools, because of their compatibilities for varied fluorometric instruments. Codon optimization has reported to increase the expression levels of several fluorescent proteins in *S. cerevisiae*^30^,^31^,^32^. However, the subjects of investigation were limited to the EGFP or other major color fluorescent proteins, and the choice of a suitable GFP for expression in *S. cerevisiae* is less well studied among a wide variety of GFPs.

Here, we expressed seven codon-optimized GFPs and five non-codon-optimized GFPs in *S. cerevisiae* and compared the protein expression levels and green fluorescence intensities. Through the evaluations, we determined the nucleic acid sequences of the codon-optimized GFPs that were expressed at high levels and that emitted intense green fluorescence in *S. cerevisiae*. Furthermore, we tested these GFPs that were expressed at relatively high levels for use as reporter genes in fusion form for detecting signal transduction and protein–protein interactions. Finally, we demonstrated the codon-optimized mUkG1 fusion reporter successfully increased the detection rates of target cells using flow cytometry.

## Results and Discussion

### Expression of GFPs in *S. cerevisiae*

**Figure 1:**
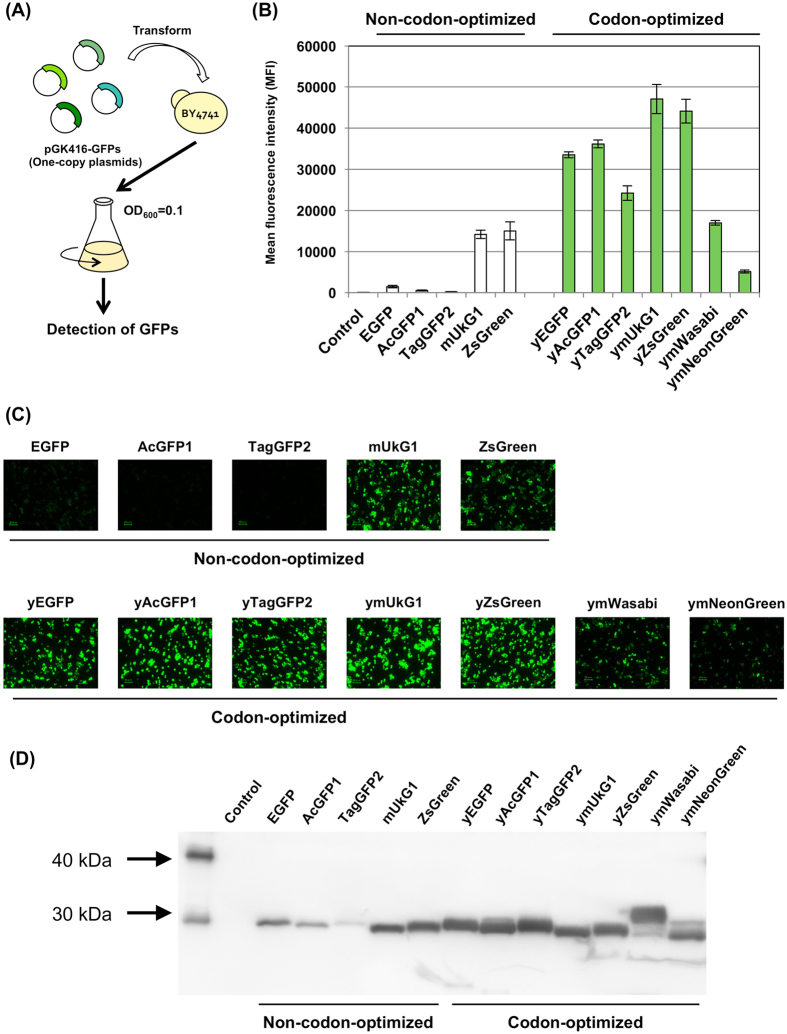
GFP expression in *S. cerevisiae*. (**A**) Flow diagram of GFP detection assays. GFP expression plasmids were transformed into the BY4741 yeast strain; the cells were then grown, and GFP expression was analyzed. (**B**) Mean GFP fluorescence intensities (MFIs). The MFIs of 10,000 cells were measured by flow cytometry. (**C**) Green fluorescence images of the cells. The images were acquired with a fluorescence microscope equipped with a 60× objective lens. Scale bar: 20 μm. The exposure time was 1/15 s. (**D**) Western blot analysis. Western blot analysis was performed using as the primary antibody monoclonal anti-frag M2 antibody for the GFPs fused frag tag. Alkaline phosphatase-conjugated anti-mouse IgG was used as the secondary antibody, and colorimetric detection of alkaline phosphatase activity was performed using CDP-Star detection reagent. ‘Control’ indicates the BY4741 yeast strain harboring a mock pGK416 plasmid.

We compared the expression of seven GFPs from various sources in *S. cerevisiae*: EGFP, AcGFP1, TagGFP2, mUkG1, ZsGreen, mWasabi and mNeonGreen (Table 1). The genes encoding the seven GFPs were optimized for expression in the yeast *S. cerevisiae* (yEGFP, yAcGFP1, yTagGFP2, ymUkG1, yZsGreen, ymWasabi and ymNeonGreen) (Supplementary Table S1) and inserted into the multiple-cloning site of a pGK416 single-copy autonomous replicating plasmid for constitutive expression under the control of the *PGK1* promoter^33^ (Fig. 1A and Table 2). The yeast BY4741 strain was transformed with the constructed plasmids (Tables 2 and 3 and Supplementary Table S2), and their green fluorescence was evaluated using flow cytometry and fluorescence microscopy (Fig. 1 and Supplementary Table S3).

The green fluorescence intensities of 10,000 cells were quantitatively measured using flow cytometry, and the values were recorded as the mean fluorescence intensities (MFIs). Monomeric ymUkG1 and tetrameric yZsGreen exhibited the highest fluorescence intensities over 40,000–45,000 MFIs (Fig. 1B and Supplementary Table S3). In contrast, ymWasabi and ymNeonGreen exhibited remarkably low fluorescence intensities (less than 20,000 MFIs) (Fig. 1B and Supplementary Table S3), even though both GFPs are the brightest green fluorescent proteins (mWasabi, ε = 70,000 M^−1^cm^−1^, QY = 0.80; mNeonGreen, ε = 116,000 M^−1^cm^−1^, QY = 0.80)^24^,^29^ (Table 1). Fluorescence microscopy observations also showed that the green fluorescence of these two proteins was less intense than that of the other five GFPs (Fig. 1C). Therefore, we decided to use GFPs other than mWasabi and mNeonGreen (EGFP, AcGFP1, TagGFP2, mUkG1 and ZsGreen) in the later experiments.

Next, we compared the expression of the codon-optimized sequences and the commercially available (non-optimized for *S. cerevisiae*) sequences of five GFPs (Table 2 and Supplementary Table S2) by flow cytometry, fluorescence microscopy and western blot analysis (Fig. 1B–D). Of the commercially available GFP sequences tested, EGFP, AcGFP1 and TagGFP2 include codons that were optimized for mammalian cell expression, and ZsGreen and mUkG1 used the codons from the isolated, wild type organism. The flow cytometry and fluorescence microscopy observations for codon-optimized GFPs are shown in Fig. 1B,C. mUkG1 and ZsGreen (non-codon-optimized), including the natural codons of the soft coral *Sarcophyton* sp. and the button polyp coral *Zoanthus* sp., exhibited good fluorescence intensities (mUkG1, MFI = 14,194; ZsGreen, MFI = 15,019) (Fig. 1B and Supplementary Table S3). The MFIs of codon-optimized GFPs (ymUkG1, MFI = 47,088; yZsGreen, MFI = 44,154) were almost three-fold greater than those of non-codon-optimized sequences (Fig. 1B, Supplementary Table S3 and Fig. S1). In contrast, EGFP, AcGFP1 and TagGFP2, which were not codon-optimized for *S. cerevisiae* (these were optimized for mammalian cells) unexpectedly exhibited considerably lower fluorescence intensities (EGFP, MFI = 1,490; AcGFP1, MFI = 542; TagGFP2, MFI = 240) (Fig. 1B, Supplementary Table S3 and Fig. S1). The MFIs of these GFPs, when codon-optimized for *S. cerevisiae* (yEGFP, MFI = 33,351; yAcGFP1, MFI = 36,177; yTagGFP2, MFI = 24,250) were surprisingly 22-, 67- and 101-fold higher, respectively, than those of corresponding non-codon-optimized GFPs (Fig. 1B, Supplementary Table S3 and Fig. S1). Fluorescence microscopy revealed clear differences in the green fluorescence intensity of the codon-optimized and non-codon-optimized GFPs (Fig. 1C). Using the FLAG tag fused GFPs (Table 2 and Supplementary Table S2), the western blot analysis showed increased expression levels of the codon-optimized GFPs (approx. 27 kDa) in *S. cerevisiae* (Fig. 1D). Moreover, intriguingly, all yeast cells expressing codon-optimized GFPs generated light green colors under natural light, unlike non-codon-optimized EGFP, AcGFP1 and TagGFP2, which were never green under natural light (Supplementary Fig. S2).

As shown in Table 4, codon-optimized GFPs for *S. cerevisiae* presented high codon adaptation indexes (CAIs; 0.87~0.90) and moderate GC contents (39.7~40.4%). In contrast, commercially available, non-codon-optimized EGFP, AcGFP1 and TagGFP2 sequences unexpectedly exhibited much lower CAIs (0.50~0.56) and higher GC contents (59.0~63.6%) (Table 4), although the codon usage had been optimized for mammalian expression. This may have misled us into thinking that the mammalian cells had reasonably similar codon usages to the eukaryotic organism *S. cerevisiae*. For mUkG1 and ZsGreen, the wild type codons from the soft coral *Sarcophyton* sp. and the button polyp coral *Zoanthus* sp. were used, unexpectedly resulting in a relatively similar codon usage to that in yeast (CAIs = 0.69~0.70 and GC contents = 44.9~45.0%) (Table 4). These support our previous result that ZsGreen (original codons) exhibited more intense fluorescence than EGFP (non-codon-optimized) when used as a fluorescent reporter protein^34^. Among the codon-optimized GFPs tested in this study, monomeric ymUkG1 and tetrameric yZsGreen exhibited the most intense green fluorescence in *S. cerevisiae* (Fig. 1B).

### GFP expression when used as fusion-tagged proteins to report the activation of signal transduction in *S. cerevisiae*

**Figure 2:**
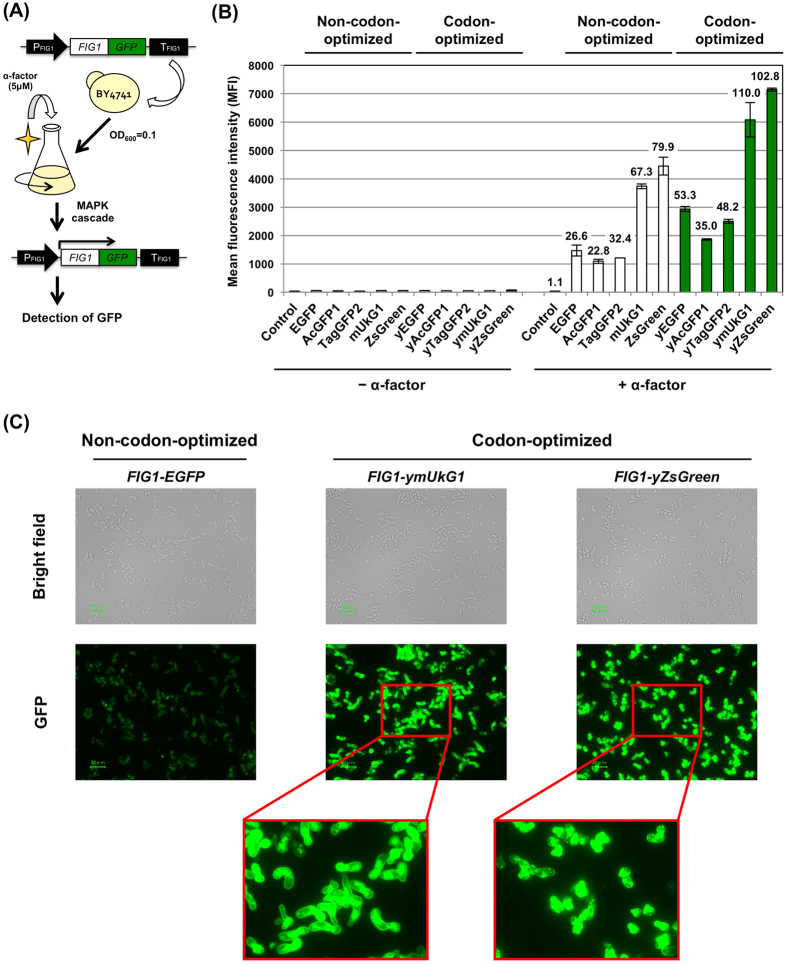
Expression of GFPs as fusion-tagged proteins to report the activation of signal transduction in *S. cerevisiae*. (**A**) Flow diagram of GFP transcription assays used to detect signal transduction. *FIG1-GFP* fusion genes were substituted for the *FIG1* gene in the yeast genome. The cells were grown in media with and without 5 μM α-factor, and GFP expression was measured. (**B**) Mean GFP fluorescence intensities (MFIs). The MFIs of 10,000 cells were measured by flow cytometry. (**C**) Green fluorescence images of the cells. The images were acquired with a fluorescence microscope equipped with a 60× objective lens. Scale bar: 20 μm. The exposure time was 1/4 s. Codon-optimized and non-codon-optimized GFPs were evaluated. ‘Control’ indicates the BY4741 wild-type yeast strain.

GFP is most widely applied as a protein tag or as a reporter gene^4^,^7^,^32^,^35^,^36^. To determine the applicability of the GFPs tested above, we decided to test the pheromone-responsive *FIG1* gene, which is involved in conjugation and cellular fusion on plasma membrane during the mating process^37^. *FIG1* gene expression is induced in response to activation of the yeast mating signaling pathway; therefore, this gene has been often used to sense the signal promoted in the mating pathway (Fig. 2A) or the agonist response to heterologous GPCRs using the non-codon-optimized *EGFP* as the reporter gene^38^,^39^,^40^,^41^.

The five codon-optimized and non-codon-optimized *GFP* genes were integrated into the chromosome of BY4741 haploid yeast **a**-cells to fuse to the C-terminus of the genomic *FIG1* gene (Table 3 and Supplementary Table S4). To induce the transcription of *FIG1-GFPs*, the cells were incubated for 6 hours in YPD medium containing the **a**-cell-specific mating pheromone (α-factor) (Fig. 2A). The cultured cells were then analyzed by flow cytometry and observed under a fluorescence microscope (Fig. 2B,C).

The GFPs tested as fusion reporters exhibited marked increases in green fluorescence in response to the addition of α-factor pheromone (Fig. 2B). Although these fusion proteins showed lower fluorescence intensities than those of the solely expressed untagged GFPs, the MFIs of the fusion-codon-optimized GFPs were greater than those of all five non-codon-optimized GFPs (Fig. 2B). The pheromone-stimulated cells induced 22.8~110.0-fold changes in the MFIs compared to the corresponding unstimulated cells (Fig. 2B). The MFIs of Fig1-GFPs were increased approximately 1.6~2.0-fold due to codon optimization. Codon-optimized ymUkG1 and yZsGreen exhibited higher fluorescence intensities than the most commonly used non-codon-optimized EGFP when expressed as fusion genes with the *FIG1* reporter gene (*FIG1-EGFP*, MFI = 1,469; *FIG1-ymUkG1*, MFI = 6,184; *FIG1-yZsGreen*, MFI = 7,143) and exhibited greater than 4.2- and 4.9-fold increases (Fig. 2B). Fluorescence microscope observation revealed brighter green fluorescence in pheromone-stimulated yeast cells encoding Fig1-ymUkG1 and Fig1-yZsGreen than in cells encoding Fig1-EGFP (the cell shapes were elongated by the excessive pheromone stimulation) (Fig. 2C). In addition, Fig1-ymUkG1 fusion protein (monomeric GFP fusion) especially showed correct localization on the plasma membrane, although it has been also observed in other organelle caused by the extensive overexpression (Fig. 2C).

As shown above, similarly to the results obtained for the solely expressed untagged GFPs, codon-optimized ymUkG1 and yZsGreen exhibited the brightest fluorescence, even when expressed as fusions with the *FIG1* reporter gene. Tetrameric fluorescent proteins are generally regarded as unfavorable for use as fusion tags because they interfere with the normal function of the target proteins^4^; however, tetrameric yZsGreen could function as the quantitative fusion reporter, although the fusion with the Fig1 protein rarely localized on the plasma membrane. In further experiments, we studied monomeric ymUkG1, which is apparently less constraining in many applications but exhibits significantly more intense fluorescence than other monomeric GFPs in *S. cerevisiae*.

### Application of codon-optimized ymUkG1 in Gγ recruitment systems that report protein–protein interactions in *S. cerevisiae*

We developed the Gγ recruitment system to detect protein–protein interactions (PPIs)^40^,^42^,^43^,^44^,^45^ and to screen for protein variants presenting desirable affinities^40^. The detection method of the Gγ recruitment system is based on the fundamental principle that yeast pheromone (mating) signaling requires the localization of a complex between guanine nucleotide binding protein (G-protein) β- and γ-subunits (Gβγ) and the inner leaflet of the plasma membrane^46^. In brief, for the machinery to detect PPIs, an engineered Gγ mutant (termed Gγ_cyto_) is used that lacks a membrane localization sequence (lipidation motif); this localization signal is normally expressed in the cytosol. When the target protein (X) is a soluble cytosolic protein, Gγ_cyto_ is prepared as a fusion protein with the target protein (X) (Gγ_cyto_-X), and library proteins (Y) are attached to the artificial lipidation site to localize the proteins to the membrane (Supplementary Fig. S3A)^40^,^42^. In contrast, when the target protein (X) is a membrane-bound protein, Gγ_cyto_ is prepared as a fused protein with the library proteins (Y) (Gγ_cyto_-Y) (Supplementary Fig. S3B)^43^,^45^. When target “X” and candidate “Y” interact, the Gγ_cyto_-X (Supplementary Fig. S3A) or Gγ_cyto_-Y (Supplementary Fig. S3B) fusion protein targets the Gβ to the membrane and subsequently induces pheromone-signaling pathway activation. Signaling can be monitored by a fluorescent reporter assay or a mating growth assay; therefore, the PPIs are easily detected (Supplementary Fig. S3C).

Here, *FIG1–EGFP* (non-optimized) was used as a fluorescent reporter gene in the Gγ recruitment system. If the flow cytometer (cell sorter) that can analyze more than several thousand cells per second is available, the fluorescent reporter assay extremely speeds up and is helpful for screening PPI protein variants. We tested whether *FIG1-ymUkG1* could improve the fluorescence reporting ability of the Gγ recruitment system. As for the previously described system, the Fc protein of human immunoglobulin G (IgG) and the Z domain (derived from *Staphylococcus aureus* protein A (Z_WT_))^47^ were used as the PPI models. Several Z variants (Z_K35A_, Z_I31A_ and Z_955_) with differing affinities for the Fc protein were also used as the PPI models (Z_WT_, 5.9 × 10^7^ M^−1^; Z_K35A_, 4.6 × 10^6^ M^−1^; Z_I31A_, 8.0 × 10^3^ M^−1^; Z_955_, none)^48^,^49^.

**Figure 3:**
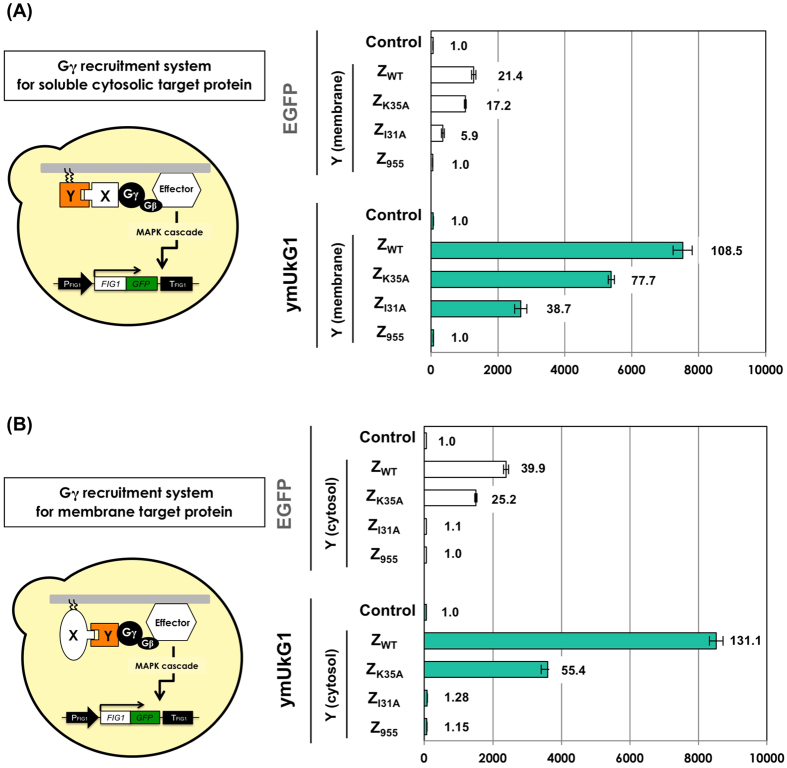
Expression of non-codon-optimized EGFP and codon-optimized ymUkG1 as fusion-tagged proteins to report PPIs using Gγ recruitment systems. (**A**) Flow cytometry analyses using the Gγ recruitment system for cytosolic target proteins. The Fc protein was used as the cytosolic target protein ‘X’ and was expressed as a fusion protein with Gγ_cyto_ (Gγ_cyto_-Fc). Membrane-anchored Z variants (Z_WT_, Z_K35A,_, Z_I31A_ and Z_955_) were expressed as ‘Y’ library candidate proteins. ‘Control’ indicates BFG2118 and UGFG2 yeast strains harboring the pGK413 mock plasmid (without the expression of ‘Y’). (**B**) Flow cytometry analyses using the Gγ recruitment system for membrane protein targets. The Fc protein was used as the membrane target protein ‘X’ and was expressed as a membrane-associated protein with the C-terminal lipid anchor (derived from Ste18p). Four Z variants (Z_WT_, Z_K35A,_, Z_I31A_ and Z_955_) were used as cytosolic candidate ‘Y’ proteins and were expressed as fusion proteins with Gγ_cyto_ (Gγ_cyto_-Z variants). ‘Control’ indicates the FC-G0 and UG2-FCG0 yeast strains (without the expression of ‘Gγ_cyto_-Y’). The engineered strains were grown in media containing 5 μM α-factor, and mean fluorescence intensities (MFIs) were analyzed. The MFIs of 10,000 cells were measured by flow cytometry.

We first tested the use of soluble cytosolic target proteins (Supplementary Fig. S3A). The Fc protein was used as the cytosolic target protein ‘X’ and was fused with Gγ_cyto_ (Gγ_cyto_-Fc). Four Z domain variants were attached to the artificial lipidation motifs (derived from Ste18 p) as the candidate ‘Y’ proteins, which were localized on the membrane (Tables 2 and 3 and Supplementary Table S5). After the addition of α-factor pheromone, the cells were cultured, and the GFP signal was measured using flow cytometry (Supplementary Fig. S3C). Both reporters (Fig1-EGFP and Fig1-ymUkG1) successfully detected PPIs between Fc and several Z variants (Z_WT_, Z_K35A_ and Z_I31A_) (Fig. 3A). However, the fluorescence intensities of the two reporters differed greatly. When comparing the systems in Z_WT_, the Fig1-EGFP and Fig1-ymUkG1 reporters exhibited 21- and 109-fold more intense fluorescence than the respective negative control cells. The fluorescence intensity of Fig1-EGFP (MFI = 1,276) was 5.9-fold less than that of Fig1-ymUkG1 (MFI = 7,524). The differences in fluorescence intensities between the Fig1-EGFP and Fig1-ymUkG1 reporters were 5.2- and 7.6-fold for the Z_K35A_ and the Z_I31A_ variants, respectively. When using the Z variant that lacks affinity for Fc (Z_955_), green fluorescence was never observed. The ymUkG1 reporter exhibited an intense fluorescence signal (MFI = 2,682) even when detecting the weak PPI between Fc and Z_I31A_ (8.0 × 10^3^ M^−1^); in contrast, Fig1-EGFP exhibited a faint fluorescence signal (MFI = 350).

Next, we tested the use of membrane target proteins (Supplementary Fig. S3B). The Fc protein was used as the membrane target protein ‘X’. To determine the positions at which to fuse the lipidation motifs, both C-terminal and N-terminal membrane-associated Fc proteins were prepared (a C-terminal lipid anchor, derived from Ste18 p; and a N-terminal lipid anchor, derived from Gpa1p). Four Z domain variants were used as cytosolic candidate ‘Y’ proteins and fused with Gγ_cyto_ (Gγ_cyto_-Z variants) (Table 3 and Supplementary Table S6). The cells were assayed similarly to the previous experiments. For the C-terminal membrane-associated Fc proteins, both the Fig1-EGFP and Fig1-ymUkG1 reporters successfully detected the PPIs with two Z variants (Z_WT_ and Z_K35A_), although they did not detect the PPI with Z_I31A_ (Fig. 3B). This result was consistent with previous results that failed to detect a PPI between membrane-associated Fc and Gγ_cyto_-fused Z_I31A_. The weak PPI (8.0 × 10^3^ M^−1^) is likely below the detection limitation in the Gγ recruitment system when using Fc as the membrane target protein^45^. Similarly to the results of the test for the soluble cytosolic target Fc protein (Fig. 3A), a large difference was observed in the fluorescence intensities between the Fig1-EGFP and Fig1-ymUkG1 reporters (Fig. 3B). When comparing the variants in Z_WT_, the Fig1-EGFP and Fig1-ymUkG1 reporters emitted fluorescent signals that were 40- and 131-times more intense than those of the respective negative control cells. The fluorescence intensities were 3.6-fold different between Fig1-EGFP (MFI = 2,380) and Fig1-ymUkG1 (MFI = 8,519). When comparing the variants in Z_K35A_, the difference in fluorescence intensity between the Fig1-EGFP and Fig1-ymUkG1 reporters was 2.4-fold. When using the N-terminal membrane-associated Fc proteins, similar tendencies were observed (Supplementary Fig. S4). When using Z_WT_ and Z_K35A,_ the differences in fluorescence intensity between the Fig1-EGFP and Fig1-ymUkG1 reporters were 3.6- and 2.9-fold, respectively. Thus, when used as a fluorescence reporter, codon-optimized ymUkG1 yielded brighter fluorescence than the previous non-codon-optimized (for *S. cerevisiae*) EGFP, even in the Gγ recruitment systems, which detect PPIs for both soluble cytosolic target proteins and membrane target proteins.

### FACS analysis of target cells in cell mixtures containing positive and negative cells

Fluorescence activated cell sorting (FACS) is a specialized instrument that enables analysis and sorting of rare target cells in a larger cell population. To evaluate the performance of ymUkG1 as the reporter for FACS analysis, the Gγ recruitment system for soluble cytosolic target proteins was used (Supplementary Fig. S3A and Fig. 3A).

To determine the gate area that dominantly included positive cells (GFP^+^), we analyzed the data of yeast strains expressing Gγ_cyto_-Fc and membrane-anchored Z variants (Z_WT_, Z_K35A_, Z_I31A_ and Z_955_), whose fluorescence was acquired using a flow cytometer in Fig. 3A. We set the gate area (GFP^+^) that completely excluded negative cells (expressing Z_955_ with Gγ_cyto_-Fc) and determined the positive cells by making the dot plots (forward scatter, FSC-A vs green fluorescence, GFP-A) (Supplementary Fig. S5). The proportions of positive cells included in the 10,000 cells (expressing Z_WT_, Z_K35A_ and Z_I31A_ with Gγ_cyto_-Fc) using the Fig1-ymUkG1 reporter were respectively 98.3%, 95.4% and 80.3%, while those using the Fig1-EGFP reporter were 58.8%, 13.9% and 2.5% (Supplementary Fig. S5 and Table S7). This indicates the Fig1-ymUkG1 reporter clearly displayed higher percentages of the cells that were defined as positive cells (GFP^+^) than the Fig1-EGFP reporter, even though both cells expressing the same interaction pairs could be presumed to coincide in the promoted signaling and GFP transcription levels.

**Figure 4:**
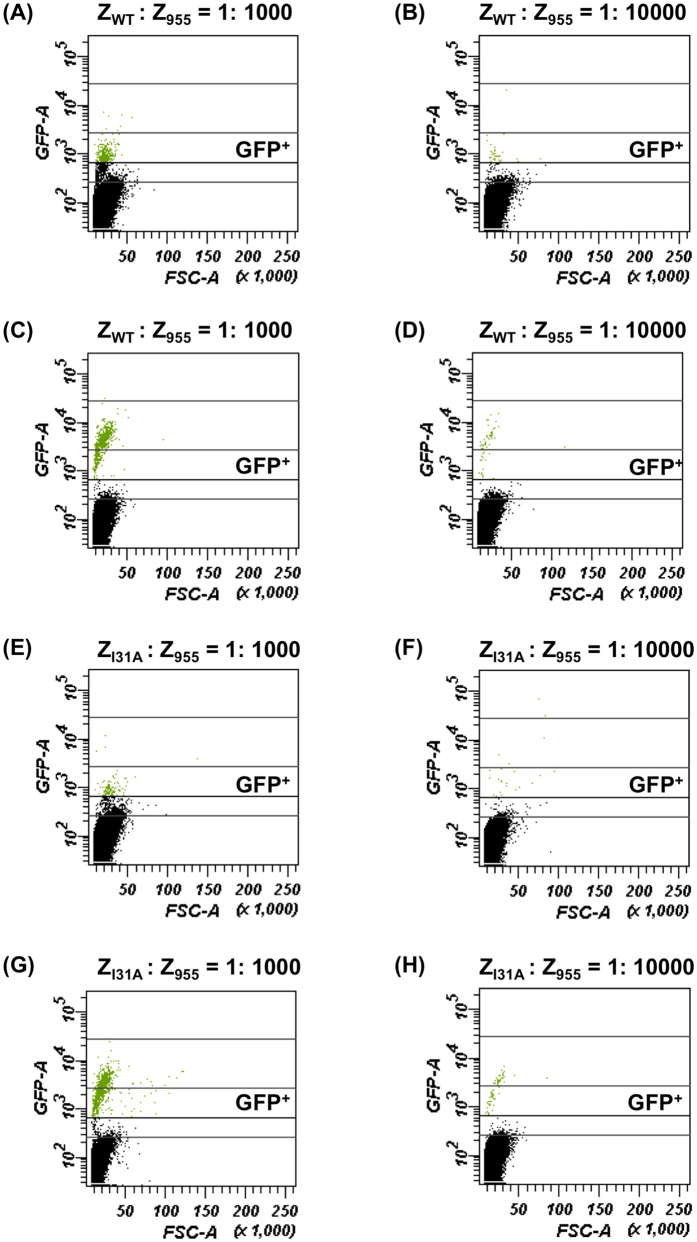
FACS detections of target cells in cell mixtures containing positive and negative cells. Data were presented as dot plots (forward scatter, FSC-A vs green fluorescence, GFP-A). Y-axis is an indication of fluorescence and X-axis is an approximation of relative cell size. Cell mixtures were prepared by mixing positive cells (expressing membrane-anchored Z_WT_ or Z_I31A_ with Gγ_cyto_-Fc) and negative cells (expressing membrane-anchored Z_955_ with Gγ_cyto_-Fc) with two different mixing rates (0.1% and 0.01% positive cells). After cultivation of the cell mixtures in the presence of α-factor for 6 hours, 10^6^ cells were analyzed using flow cytometer and the numbers of the positive (target) cells contained in the gate area (GFP^+^) were evaluated. (**A,B**) Cell mixtures containing 0.1% and 0.01% target cells expressing Z_WT_ in BFG2118 yeast strain (EGFP reporter). (**C,D**) Cell mixtures containing 0.1% and 0.01% target cells expressing Z_WT_ in UGFG2 yeast strain (ymUkG1 reporter). (**E,F**) Cell mixtures containing 0.1% and 0.01% target cells expressing Z_I31A_ in BFG2118 yeast strain (EGFP reporter). (**G,H**) Cell mixtures containing 0.1% and 0.01% target cells expressing Z_I31A_ in UGFG2 (ymUkG1 reporter).

To further demonstrate the ability of the ymUkG1 reporter, we prepared the cell mixtures composed of positive cells (expressing Z_WT_ with Gγ_cyto_-Fc) and negative cells (expressing Z_955_ with Gγ_cyto_-Fc) with two different mixing rates (0.1% and 0.01% positive cells). After cultivation of the cell mixtures in the presence of α-factor for 6 hours, 10^6^ cells were analyzed using flow cytometer and the content rates of positive (target) cells contained in the gate area (GFP^+^) were evaluated (Fig. 4 and Table 5). Both Fig1-EGFP and Fig1-ymUkG1 reporters exposed the target cells contained in the GFP^+^ area, whereas they showed the obvious distinctions in the detection rates of the target cells (Fig. 4 and Table 5). When using the cell mixtures expressing Z_WT_ and Z_955_ with Gγ_cyto_-Fc (initially contained 0.1% and 0.01% positive cells), Fig1-ymUkG1 reporter detected 58% and 56% of the target cells (percentages of detected target cell numbers in GFP^+^ area against initial cell numbers of positive cells), while Fig1-EGFP reporter detected 33% and 34% of the target cells, respectively (Fig. 4A–D and Table 5). When using the cell mixtures expressing Z_I31A_ and Z_955_ with Gγ_cyto_-Fc (in the case of weak interaction), more clear differences were found between Fig1-EGFP and Fig1-ymUkG1 reporters (Fig. 4E–H and Table 5). Briefly, Fig1-ymUkG1 reporter detected 74% and 65% of the target cells, while Fig1-EGFP reporter detected 12% and 24% of the target cells (for the cell mixtures containing 0.1% and 0.01% positive cells, respectively) (Fig. 4E–H and Table 5). The reason why the use of Z_I31A_ displayed higher percentages of the detectable target cells than that of Z_WT_ might have arisen from the growth biases during the cultivation of cell mixtures. It is well known that the pheromone signaling (in response to the PPIs) provokes the cell-cycle arrest in G1 phase to make arrangements for the mating^50^. Because the percentages of the detectable positive cells to the initially contained positive cell numbers directly affect the recovery rates of the hopeful target cells, they are crucially important factors on FACS sorting. Thus, we have successfully demonstrated the superiority of the codon-optimized ymUkG1 reporter compared to the non-codon-optimized EGFP reporter in *S. cerevisiae*.

In summary, we compared the expression of seven codon-optimized GFPs and five non-codon-optimized GFPs in *S. cerevisiae*. Codon optimization for *S. cerevisiae* improved all five non-codon-optimized GFPs and surprisingly increased the fluorescence intensities up to 101-fold compared with commercially available GFPs (which were optimized for mammalian codon usage). CAI and GC content affected the expression of GFPs in *S. cerevisiae* more than expected. Among the codon-optimized GFPs tested, monomeric ymUkG1 and tetrameric yZsGreen exhibited the most intense fluorescence in *S. cerevisiae*, and green color was observed visually under natural light conditions in cells expressing these proteins. The expression of monomeric ymUkG1 protein was higher than that of tetrameric yZsGreen protein. Both ymUkG1 and yZsGreen were prepared as fusion reporters tagged to the Fig1 protein for use in monitoring signal transduction, and these fluorescent reporters exhibited significantly greater utility than the previously used, non-codon-optimized EGFPs (which were optimized for mammalian systems). Finally, the monomeric ymUkG1 was successfully used to improve the PPI detection system using the Fig1 reporter and increased the percentages of the detectable target cells using flow cytometry.

The most commonly used GFP in *S. cerevisiae* is probably the commercially available EGFP, whose codons are optimized for mammalian cells, not *S. cerevisiae*. Because gene and fragment synthesis services are readily available, it is easy to prepare a DNA fragment containing codon-optimized nucleic acid sequences. When strong protein expression is required, it might be best to measure the CAI and GC content and to test the codon-optimization of the protein of interest. We propose that the codon-optimized monomeric protein ymUkG1 (Supplementary Table S1) is a good choice for GFP experiments involving *S. cerevisiae*.

## Methods

### Strains and media

Details of the yeast *S. cerevisiae* strains BY4741^51^, BY4742^51^, MC-F1^44^ and other recombinant strains used in this study and their genotypes are outlined in Table 3. The yeast strains were grown in YPD medium containing 1% (w/v) yeast extract, 2% peptone and 2% glucose or in SD media containing 0.67% yeast nitrogen base without amino acids (BD-Diagnostic Systems, Sparks, MD, USA) and containing 2% glucose. Amino acids and nucleotides (20 mg/L histidine, 60 mg/L leucine, 20 mg/L methionine, or 20 mg/L uracil) were supplemented into SD media lacking the relevant auxotrophic components. Agar (2%; w⁄v) was added to the medium described above to produce YPD and SD solid media.

### Codon optimization and calculation of the codon adaptation index (CAI) and GC content

The nucleic acid sequences for codon-optimized GFPs for *S. cerevisiae* were designed using GeneArt^®^ GeneOptimizer^®^ software (Life Technologies/Thermo Fisher Scientific, San Jose, CA, USA). The DNA fragments for codon-optimized GFPs were prepared by using the GeneArt^®^ Strings^™^ DNA fragments service. Codon adaptation indexes (CAIs) and GC contents before and after optimization were determined using GenScript Rare Codon Analysis Tool software (GenScript, Piscataway, NJ, USA).

### Plasmid construction

All plasmids and primers used in this study are listed in Table 2 and Supplementary Table S8. The plasmids used for the expression of the tested GFPs were constructed as follows: DNA fragments encoding the GFPs AcGFP1, TagGFP2 and mUkG1 were PCR-amplified from pAcGFP1 (Clontech Laboratories/Takara Bio, Shiga, Japan), pTagGFP2-tubulin (Evrogen, Moscow, Russia) and pmUkG1-S1 (Medical & Biological Laboratories, Nagoya, Japan) using primer pairs 1 and 2, 3 and 4, and 5 and 6; the fragments were then digested with SalI + BamHI and inserted into the same sites between the *PGK1* promoter (*P*_*PGK1*_) and the *PGK1* terminator (*T*_*PGK1*_) on pGK416^33^, yielding the plasmids pGK416-AcGFP1, pGK416-TagGFP2 and pGK416-mUkG1. For EGFP and ZsGreen, the previously constructed plasmids pGK416-EGFP^33^ and pGK416-ZsGreen^34^ were used. The expression plasmids used for codon-optimized GFPs (yEGFP, yAcGFP1, yTagGFP2, ymUkG1, yZsGreen, ymWasabi and ymNeonGreen) were constructed as follows: DNA fragments encoding the codon-optimized GFPs were PCR-amplified from the GeneArt^®^ Strings^™^ DNA fragments using primer pairs 7 and 8, 9 and 10, 11 and 12, 13 and 14, 15 and 16, 17 and 18, and 19 and 20; the fragments were then digested with SalI + BamHI and inserted into the same sites between the *P*_*PGK1*_ and the *T*_*PGK1*_ on pGK416^33^, yielding the plasmids pGK416-yEGFP, pGK416-yAcGFP1, pGK416-yTagGFP2, pGK416-ymUkG1, pGK416-yZsGreen, pGK416-ymWasabi and pGK416-ymNeonGreen.

The plasmids used for the western blotting were constructed as follows: DNA fragments encoding the GFPs fused the FLAG tag sequence were PCR-amplified from pGK416-EGFP, pGK416-AcGFP1, pGK416-TagGFP2, pGK416-mUkG1, pGK416-ZsGreen, pGK416-yEGFP, pGK416-yAcGFP1, pGK416-yTagGFP2, pGK416-ymUkG1 and pGK416-yZsGreen using primer pairs 21 and 22, 1 and 23, 3 and 24, 5 and 25, 26 and 27, 7 and 28, 9 and 29, 11 and 30, 13 and 31, and 15 and 32, 17 and 33, and 19 and 34 respectively. Primers 22, 23, 24, 25, 27, 28, 29, 30, 31 and 32 contain FLAG tag (24 bp). Then, the fragments were then digested with SalI + BamHI and inserted into the same sites between the *P*_*PGK1*_ and the *T*_*PGK1*_ on pGK416^33^, yielding the plasmids pGK416-EGFP-F, pGK416-AcGFP1-F, pGK416-TagGFP2-F, pGK416-mUkG1-F pGK416-ZsGreen, pGK416-yEGFP-F, pGK416-yAcGFP1-F, pGK416-yTagGFP2-F, pGK416-ymUkG1-F, pGK416-yZsGreen-F, pGK416-ymWasabi-F and pGK416-ymNeonGreen-F.

The plasmids used to integrate the *GFP* reporter genes at the *FIG1* locus on the yeast chromosome were constructed as follows: A DNA fragment containing the homologous sequence of the *FIG1* terminator (downstream of *FIG1* gene; 200 bp) was PCR-amplified from pBlue-FIG1pt-ZsGreen^34^ using the primer pair 35 and 36. The amplified fragments were digested with XhoI + KpnI and inserted into the pBlueScript II KS(+) vector, yielding the plasmid pBlue-FIG1t. A DNA fragment containing the *URA3* selectable marker was PCR-amplified from pBlue-FIG1pt-ZsGreen^34^ using the primer pair 37 and 38. DNA fragments containing the GFPs were PCR-amplified from pGK416-AcGFP1, pGK416-TagGFP2, pGK416-mUkG1, pGK416-ZsGreen, pGK416-yEGFP, pGK416-yAcGFP1, pGK416-yTagGFP2, pGK416-ymUkG1, and pGK416-yZsGreen^34^ using primer pairs 39 and 40, 41 and 42, 43 and 44, 45 and 46, 47 and 48, 49 and 50, 51 and 52, 53 and 54, and 55 and 56, respectively. Primers 39, 41, 43, 45, 47, 49, 40, 53 and 55 contain regions that are homologous to the C-terminus of *FIG1* (50 bp). Then, the *URA3* fragment and the GFP fragment were then linked by overlap PCR using primer pairs 39 and 38, 41 and 38, 43 and 38, 45 and 38, 47 and 38, 49 and 38, 51 and 38, 53 and 38, and 55 and 38, and the overlap fragments were digested with SacII + XhoI and inserted into pBlue-FIG1t, yielding the plasmids pBlue-UFt-AcGFP1, pBlue-UFt-TagGFP2, pBlue-UFt-mUkG1, pBlue-UFt-ZsGreen, pBlue-UFt-yEGFP, pBlue-UFt-yAcGFP1, pBlue-UFt-yTagGFP2, pBlue-UFt-ymUkG1 and pBlue-UFt-yZsGreen.

### Yeast strain construction

The strains used in this study are listed in Table 3. DNA cassettes were integrated to express GFP-fused *FIG1* as follow: DNA fragments containing *FIG1*(50 bp)*-GFP-URA3-T*_*FIG1*_ (*T*_*FIG1*_: *FIG1* terminator) were amplified from pBlue-UFt-AcGFP1, pBlue-UFt-TagGFP2, pBlue-UFt-mUkG1, pBlue-UFt-ZsGreen, pBlue-UFt-yEGFP, pBlue-UFt-yAcGFP1, pBlue-UFt-yTagGFP2, pBlue-UFt-ymUkG1 and pBlue-UFt-yZsGreen using the primer pair 57 and 58. BY4741 was transformed with the amplified DNA fragments using the lithium acetate method^52^. The transformants were selected on SD-Ura plates (SD solid medium without uracil, but containing leucine, histidine and methionine). After confirming the correct integration, the *URA3* marker was “popped-out” by homologous recombination using counter-selection with 5-fluoroorotic acid (5-FOA, Fluorochem, Derbyshire, UK), to yield BYFAG1, BYFTG1, BYFUG1, BYFZG1, BYFEG2, BYFAG2, BYFTG2, BYFUG2 and BYFZG2.

The *STE18* gene was substituted by *kanMX4* in the yeast chromosome by amplifying the DNA fragment containing *P*_*STE18*_*–kanMX4–T*_*STE18*_ (*P*_*STE18*_: *STE18* promoter and *T*_*STE18*_: *STE18* terminator) from pGK426-GPTK^42^ using the primer pair 45 and 46. BYFUG2 was then transformed with the amplified DNA fragment, and transformants were selected on YPD solid medium containing G418 (500 ng/mL) (Nacalai Tesque, Kyoto, Japan) to yield the UGW2 strain.

DNA cassettes for the Gγ_cyto_–Fc protein in the cytosol were integrated as follows. DNA fragments containing *URA3-P*_*PGK1*_*-Gγ*_*cyto*_*-Fc-T*_*PGK1*_*-T*_*HIS3*_ (*T*_*HIS3*_: *HIS3* terminator) were amplified from pUMGP-GγMFcH^42^ using the primer pair 61 (containing the homologous regions of the *HIS3* promoter) and 62. UGW2 was transformed with the amplified DNA fragments using the lithium acetate method. The transformants were selected on SD-Ura plates (containing leucine, histidine and methionine) to yield UGFG2.

DNA cassettes used to express the membrane-associated Fc protein were integrated as follows: DNA fragments containing *P*_*STE18*_*-P*_*PGK1*_*-Fc-Ste18C-T*_*PGK1*_*-kanMX4-T*_*STE18*_ and *P*_*STE18*_*-P*_*PGK1*_*-Gpa1N-Fc-T*_*PGK1*_*-kanMX4-T*_*STE18*_ were amplified from pUMGPTK-Fc-Ste18C^45^ and pUMGPTK-Gpa1N-Fc^45^, respectively, using the primer pair 59 and 60. BYFUG2 was transformed with the amplified DNA fragments using the lithium acetate method. The transformants were selected on YPD solid medium containing G418 (500 ng/mL) to yield BYFUG2-FC and BYFUG2-FN. DNA cassettes for the Gγ_cyto_–Z domain variants (Z_WT_, Z_K35A_, Z_I31A_ and Z_955_) in the cytosol were integrated as follows: DNA fragments containing *URA3-P*_*PGK1*_*-Gγ*_*cyto*_*-Z*_*WT*_*(-Z*_*K35A*_*, -Z*_*I31A*_ and *-Z*_*955*_*)-T*_*PGK1*_*-T*_*HIS3*_ and *URA3-P*_*PGK1*_*-Gγ*_*cyto*_*-T*_*PGK1*_*-T*_*HIS3*_ were amplified from pUSTE18p-Gγcyto-ZWT(-ZK35A, -ZI31A and -Z955)-HIS3t^45^ and pUSTE18p-Gγcyto-HIS3t^45^ using the primers 61 (containing the homologous regions of the *HIS3* promoter) and 62. BYFUG2-FC and BYFUG2-FN were transformed with the amplified DNA fragments using the lithium acetate method^52^. The transformants were selected on SD-Ura plates containing leucine, histidine and methionine to yield UG2-FCGW, UG2-FCGK, UG2-FCGI, UG2-FCG9, UG2-FCG0, UG2-FNGW, UG2-FCGK, UG2-FCGI, UG2-FCG9 and UG2-FCG0.

The constructed strains were used for the assays or plasmids were introduced using the lithium acetate method. All strains and transformants used for the assays are listed in Supplementary Tables S2–S5.

### Culture conditions for GFP expression in yeast cells

To express the tested GFPs (Fig. 1A), yeast transformants (Supplementary Table S2) were grown in 5 mL of SD-Ura medium at 30 °C overnight. The cultured cells were then inoculated into 2 mL of fresh SD-Ura medium to obtain an initial optical density at 600 nm (OD_600_) of 0.1. The cells were thereafter cultivated at 30 °C for 3 hours and then harvested.

Signal transduction assays using *FIG1-GFP* reporter genes (Fig. 2A) were conducted according to previously described methods^40^ with some modifications. The engineered yeast **a**-cells (Supplementary Table S3) were grown in 5 mL of YPD medium 30 °C overnight. The cultured cells were then inoculated into 2 mL of fresh YPD medium containing 5 μM α-factor (Zymo Research, Orange, CA, USA) to obtain an initial OD_600_ of 0.1. The cells were thereafter cultivated at 30 °C for 6 hours and then harvested.

Protein–protein interaction assays using *FIG1-GFP* reporter genes were conducted as described previously^40^. In brief, for the Gγ recruitment system involving soluble cytosolic target protein (Fig. 3A), the engineered yeast **a**-cells (Supplementary Table S4) were grown in 5 mL of SD-His medium at 30 °C overnight. The cultured cells were then inoculated into 2 mL of fresh SD-His medium containing 5 μM α-factor to obtain an initial OD_600_ of 0.1. For the Gγ recruitment system involving membrane target proteins (Fig. 3B and Supplementary Fig. S4), the engineered yeast **a**-cells (Supplementary Table S5) were grown in 5 mL of YPD medium at 30 °C overnight. The cultured cells were then inoculated in 2 mL of fresh YPD medium containing 5 μM α-factor to obtain an initial OD_600_ of 0.1. In both cases, the cells were thereafter cultivated at 30 °C for 6 hours and then harvested.

### Fluorescence microscopy

Fluorescence microscopy observation was performed as described previously^53^. The harvested cells were washed and resuspended in distilled water; the cell suspensions were then observed using a BIOREVO BZ-9000 fluorescence microscope equipped with a 60× objective lens (Keyence, Osaka, Japan). Green fluorescence images were acquired with a 470/40 band-pass filter for excitation and a 535/50 band-pass filter for emission.

### Flow cytometry

Flow cytometry analysis was conducted as described previously^54^. The harvested cells were diluted into test tubes containing the sheath solution, and GFP fluorescence was measured by using a BD FACSCanto II flow cytometer (BD Biosciences, San Jose, CA). The green fluorescence signal from 10,000 cells was excited with a 488-nm blue laser and collected through a 530/30-nm band-pass (GFP) filter. The data were analyzed by using BD FACSDiva software (version 5.0, BD Biosciences) and FlowJo software version 7.2.2 (Treestar, Inc., San Carlos, CA).

### FACS analysis of target cells in cell mixtures

To evaluate the Fig1-EGFP and Fig1-ymUkG1 reporters (Supplementary Fig. S5), yeast transformants (Supplementary Table S4) were grown in 5 mL of SD-His medium at 30 °C overnight. Cell mixtures were prepared by mixing target yeast cells (BFG2118 + pGK413-ZWTmem, UGFG2 + pGK413-ZWTmem, BFG2118 + pGK413-ZI31Amem or UGFG2 + pGK413-ZI31Amem) and negative yeast cells (BFG2118 + pGK413-Z955mem or UGFG2 + pGK413-Z955mem) with 0.1% and 0.01% of the initial target cell ratios. The cell mixtures were then inoculated into 2 mL of fresh SD-His medium containing 5 μM α-factor to obtain an initial OD_600_ of 0.1. The cells were thereafter cultivated at 30 °C for 6 hours, and then 10^6^ cells were acquired by using a BD FACSCanto II flow cytometer and analyzed by using BD FACSDiva software.

### Western blot analysis

After yeast transformants (Supplementary Table S2) were grown in 5 mL of SD-Ura medium at 30 °C for 16 hours, samples were prepared using an alkaline lysis method^55^. Protein extracts (3 μl) were resolved in a 15% e-PAGEL (Atto Co., Tokyo, Japan). Western blot analysis was performed using the monoclonal anti-FLAG M2 antibody (Sigma–Aldrich, Irvine, UK) for the GFPs fused FLAG tag. Alkaline phosphatase-conjugated anti-mouse IgG (Promega, Madison, WI, USA) was used as the secondary antibody, and colorimetric detection of alkaline phosphatase activity was performed using CDP-Star detection reagent (GE Healthcare Life Sciences, UK).

## Additional Information

**How to cite this article**: Kaishima, M. *et al*. Expression of varied GFPs in *Saccharomyces cerevisiae*: codon optimization yields stronger than expected expression and fluorescence intensity. *Sci. Rep*. **6**, 35932; doi: 10.1038/srep35932 (2016).

**Publisher’s note**: Springer Nature remains neutral with regard to jurisdictional claims in published maps and institutional affiliations.

## Supplementary Material

Supplementary Information

## Figures and Tables

**Table 1:** Properties of green fluorescent proteins tested.

Protein	λex (nm)	λem (nm)	EC (ε) (M^−1^cm^−1^)	QY	RB (% of EGFP)	Association state	Reference or source
EGFP	488	507	56,000	0.60	100	Monomer	18
AcGFP1	475	505	32,500	0.82	79	Monomer	Clontech
TagGFP2	483	506	56,500	0.60	101	Monomer	Evrogen
mUkG1	483	499	60,000	0.72	129	Monomer	26
ZsGreen	493	505	43,000	0.91	116	Tetramer	27
mWasabi	493	509	70,000	0.80	167	Monomer	29
mNeonGreen	506	517	116,000	0.80	276	Monomer	24

Along with the common name for each GFP, the peak excitation (λex) and emission (λem) wavelengths, molar extinction coefficient (EC), quantum yield (QY), relative brightness (RB), association state and reference or source are listed. Brightness values were derived from the product of EC and QY, divided by the value for EGFP.

**Table 2:** List of plasmids used in this study.

Plasmids	Genotype	Reference or source
pGK416	Expression vector containing *PGK1* promoter, *CEN/ARS* single-copy origin and *URA3* marker	33
pGK416-EGFP	EGFP expression, in pGK416	33
pGK416-AcGFP1	AcGFP1 expression, in pGK416	This study
pGK416-TagGFP2	TagGFP2 expression, in pGK416	This study
pGK416-mUkG1	mUkG1 expression, in pGK416	This study
pGK416-ZsGreen	ZsGreen expression, in pGK416	34
pGK416-yEGFP	yEGFP (codon-optimized EGFP) expression, in pGK416	This study
pGK416-yAcGFP1	yAcGFP1 (codon-optimized AcGFP1) expression, in pGK416	This study
pGK416-yTagGFP2	yTagGFP2 (codon-optimized TagGFP2) expression, in pGK416	This study
pGK416-ymUkG1	ymUkG1 (codon-optimized mUkG1) expression, in pGK416	This study
pGK416-yZsGreen	yZsGreen (codon-optimized ZsGreen) expression, in pGK416	This study
pGK416-ymWasabi	ymWasabi (codon-optimized mWasabi) expression, in pGK416	This study
pGK416-ymNeonGreen	ymNeonGreen (codon-optimized mNeonGreen) expression, in pGK416	This study
pGK416-EGFP-F	EGFP-FLAG expression, in pGK416	This study
pGK416-AcGFP1-F	AcGFP1-FLAG expression, in pGK416	This study
pGK416-TagGFP2-F	TagGFP2-FLAG expression, in pGK416	This study
pGK416-mUkG1-F	mUkG1-FLAG expression, in pGK416	This study
pGK416-ZsGreen-F	ZsGreen-FLAG expression, in pGK416	This study
pGK416-yEGFP-F	yEGFP (codon-optimized EGFP) -FLAG expression, in pGK416	This study
pGK416-yAcGFP1-F	yAcGFP1 (codon-optimized AcGFP1) -FLAG expression, in pGK416	This study
pGK416-yTagGFP2-F	yTagGFP2 (codon-optimized TagGFP2) -FLAG expression, in pGK416	This study
pGK416-ymUkG1-F	ymUkG1 (codon-optimized mUkG1) -FLAG expression, in pGK416	This study
pGK416-yZsGreen-F	yZsGreen (codon-optimized ZsGreen)-FLAG expression, in pGK416	This study
pGK416-ymWasabi-F	ymWasabi (codon-optimized mWasabi)-FLAG expression, in pGK416	This study
pGK416-ymNeonGreen-F	ymNeonGreen (codon-optimized mNeonGreen)-FLAG expression, in pGK416	This study
pBlueScript II KS(+)	Cloning vector	Agilent Technologies
pBlue-FIG1pt-ZsGreen	*P*_*FIG1*_ *(300 bp)-URA3-ZsGreen-T*_*FIG1*_ *(200 bp)* in pBlueScript II KS(+)	34
pBlue-FIG1t	*T*_*FIG1*_ *(200 bp)* in pBlueScript II KS(+)	This study
pBlue-UFt-AcGFP1	*C-terminus of FIG1 (50 bp)-AcGFP1-URA3-T*_*FIG1*_ *(200 bp)* in pBlueScript II KS(+)	This study
pBlue-UFt-TagGFP2	*C-terminus of FIG1 (50 bp)-TagGFP2-URA3-T*_*FIG1*_ *(200 bp)* in pBlueScript II KS(+)	This study
pBlue-UFt-mUkG1	*C-terminus of FIG1 (50 bp)-mUkG1-URA3-T*_*FIG1*_ *(200 bp)* in pBlueScript II KS(+)	This study
pBlue-UFt-ZsGreen	*C-terminus of FIG1 (50 bp)-ZsGreen-URA3-T*_*FIG1*_ *(200 bp)* in pBlueScript II KS(+)	This study
pBlue-UFt-yEGFP	*C-terminus of FIG1 (50 bp)-yEGFP-URA3-T*_*FIG1*_ *(200 bp)* in pBlueScript II KS(+)	This study
pBlue-UFt-yAcGFP1	*C-terminus of FIG1 (50 bp)-yAcGFP1-URA3-T*_*FIG1*_ *(200 bp)* in pBlueScript II KS(+)	This study
pBlue-UFt-yTagGFP2	*C-terminus of FIG1 (50 bp)-yTagGFP2-URA3-T*_*FIG1*_ *(200 bp)* in pBlueScript II KS(+)	This study
pBlue-UFt-ymUkG1	*C-terminus of FIG1 (50 bp)-ymUkG1-URA3-T*_*FIG1*_ *(200 bp)* in pBlueScript II KS(+)	This study
pBlue-UFt-yZsGreen	*C-terminus of FIG1 (50 bp)-yZsGreen-URA3-T*_*FIG1*_ *(200 bp)* in pBlueScript II KS(+)	This study
pGK426	Expression vector containing *PGK1* promoter, 2 *μ* origin and *URA3* marker	33
pGK426-GPTK	*URA3-P*_*STE18*_*-kanMX4-T*_*STE18*_ in pGK426	42
pUSTE18p-Gγcyto-HIS3t	*URA3-P*_*STE18*_*-Gγ*_*cyto*_*-T*_*PGK1*_*-T*_*HIS3*_ in pGK426	45
pUMGP-GγMFcH	*URA3-P*_*STE18*_*-Gγ*_*cyto*_*-Fc-T*_*PGK1*_ in pUSTE18p-Gγcyto-HIS3t	42
pGK413	Expression vector containing *PGK1* promoter, *CEN/ARS* single-copy origin and *HIS3* marker	33
pGK413-ZWTmem	Z_WT_ and C-terminus of Ste18 (9 a.a.) fusion expression, in pGK413	40
pGK413-ZK35Amem	Z_K35A_ and C-terminus of Ste18 (9 a.a.) fusion expression, in pGK413	40
pGK413-ZI31Amem	Z_I31A_ and C-terminus of Ste18 (9 a.a.) fusion expression, in pGK413	40
pGK413-Z955mem	Z_955_ and C-terminus of Ste18 (9 a.a.) fusion expression, in pGK413	40
pGK426-GPTK	*URA3-P*_*STE18*_*-kanMX4-T*_*STE18*_ in pGK426	42
pUMGPTK-Gpa1N-Fc	*URA3-P*_*STE18*_*-P*_*PGK1*_*-Gpa1N (9 a.a.)-Fc-T*_*PGK1*_*-kanMX4-T*_*STE18*_ in pGK426-GPTK	45
pUMGPTK-Fc-Ste18C	*URA3-P*_*STE18*_*-P*_*PGK1*_*-Fc-Ste18C (9 a.a.)-T*_*PGK1*_*-kanMX4-T*_*STE18*_ in pGK426-GPTK	45
pUSTE18p-Gγcyto-HIS3t	*URA3-P*_*STE18*_*-Gγ*_*cyto*_*-T*_*PGK1*_*-T*_*HIS3*_ in pGK426	45
pUSTE18p-Gγcyto-ZWT-H	*URA3-P*_*STE18*_*-Gγ*_*cyto*_*-Z*_*WT*_*-T*_*PGK1*_ in pUSTE18p-Gγcyto-HIS3t	45
pUSTE18p-Gγcyto-ZK35A-H	*URA3-P*_*STE18*_*-Gγ*_*cyto*_*-Z*_*K35A*_*-T*_*PGK1*_ in pUSTE18p-Gγcyto-HIS3t	45
pUSTE18p-Gγcyto-ZI31A-H	*URA3-P*_*STE18*_*-Gγ*_*cyto*_*-Z*_*I31A*_*-T*_*PGK1*_ in pUSTE18p-Gγcyto-HIS3t	45
pUSTE18p-Gγcyto-Z955-H	*URA3-P*_*STE18*_*-Gγ*_*cyto*_*-Z*_*955*_*-T*_*PGK1*_ in pUSTE18p-Gγcyto-HIS3t	45

**Table 3:** Yeast strains used in this study.

Strain	Relevant feature	Reference
BY4741	*MAT*a *his3*Δ*1 ura3*Δ*0 leu2*Δ*0 met15*Δ*0*	51
BY4742	*MAT*α *his3*Δ*1 ura3*Δ*0 leu2*Δ*0 lys2*Δ*0*	51
MC-F1	BY4741 *fig1*::*FIG1-EGFP*	44
BYFAG1	BY4741 *fig1*::*FIG1-AcGFP1*	This study
BYFTG1	BY4741 *fig1*::*FIG1-TagGFP2*	This study
BYFUG1	BY4741 *fig1*::*FIG1-mUkG1*	This study
BYFZG1	BY4741 *fig1*::*FIG1-ZsGreen*	This study
BYFEG2	BY4741 *fig1*::*FIG1-yEGFP (codon optimized EGFP)*	This study
BYFAG2	BY4741 *fig1*::*FIG1-yAcGFP1 (codon optimized AcGFP1)*	This study
BYFTG2	BY4741 *fig1*::*FIG1-yTagGFP2 (codon optimized TagGFP2)*	This study
BYFUG2	BY4741 *fig1*::*FIG1-ymUkG1 (codon optimized mUkG1)*	This study
BYFZG2	BY4741 *fig1*::*FIG1-yZsGreen (codon optimized ZsGreen)*	This study
BFG2118	MC-F1 *ste18*Δ::*kanMX4 his3*Δ::*URA3-P*_*STE18*_*-Gγ*_*cyto*_*-Fc*	42
UGW2	BYFUG2 *ste18*Δ::*kanMX4*	This study
UGFG2	BYFUG2 *ste18*Δ::*kanMX4 his3*Δ::*URA3-P*_*STE18*_*-Gγ*_*cyto*_*-Fc*	This study
MC-FC	MC-F1 *ste18*Δ::*kanMX4-P*_*PGK1*_*-Fc-Ste18C*	45
MC-FN	MC-F1 *ste18*Δ::*kanMX4-P*_*PGK1*_*-Gpa1N-Fc*	45
FC-GW	MC-F1 *ste18*Δ::*kanMX4-P*_*PGK1*_*-Fc-Ste18C his3*Δ::*URA3-P*_*STE18*_*-Gγ*_*cyto*_*-Z*_*WT*_	45
FC-GK	MC-F1 *ste18*Δ::*kanMX4-P*_*PGK1*_*-Fc-Ste18C his3*Δ::*URA3-P*_*STE18*_*-Gγ*_*cyto*_*-Z*_*K35A*_	45
FC-GI	MC-F1 *ste18*Δ::*kanMX4-P*_*PGK1*_*-Fc-Ste18C his3*Δ::*URA3-P*_*STE18*_*-Gγ*_*cyto*_*-Z*_*I31A*_	45
FC-G9	MC-F1 *ste18*Δ::*kanMX4-P*_*PGK1*_*-Fc-Ste18C his3*Δ::*URA3-P*_*STE18*_*-Gγ*_*cyto*_*-Z*_*955*_	45
FC-G0	MC-F1 *ste18*Δ::*kanMX4-P*_*PGK1*_*-Fc-Ste18C his3*Δ::*URA3*	45
FN-GW	MC-F1 *ste18*Δ::*kanMX4-P*_*PGK1*_*-Gpa1N-Fc his3*Δ::*URA3-P*_*STE18*_*-Gγ*_*cyto*_*-Z*_*WT*_	45
FN-GK	MC-F1 *ste18*Δ::*kanMX4-P*_*PGK1*_*-Gpa1N-Fc his3*Δ::*URA3-P*_*STE18*_*-Gγ*_*cyto*_*-Z*_*K35A*_	45
FN-GI	MC-F1 *ste18*Δ::*kanMX4-P*_*PGK1*_*-Gpa1N-Fc his3*Δ::*URA3-P*_*STE18*_*-Gγ*_*cyto*_*-Z*_*I31A*_	45
FN-G9	MC-F1 *ste18*Δ::*kanMX4-P*_*PGK1*_*-Gpa1N-Fc his3*Δ::*URA3-P*_*STE18*_*-Gγ*_*cyto*_*-Z*_*955*_	45
FN-G0	MC-F1 *ste18*Δ::*kanMX4-P*_*PGK1*_*-Gpa1N-Fc his3*Δ::*URA3*	45
BYFUG2-FC	BYFUG2 *ste18*Δ::*kanMX4-P*_*PGK1*_*-Fc-Ste18C*	This study
BYFUG2-FN	BYFUG2 *ste18*Δ::*kanMX4-P*_*PGK1*_*-Gpa1N-Fc*	This study
UG2-FCGW	BYFUG2 *ste18*Δ::*kanMX4-P*_*PGK1*_*-Fc-Ste18C his3*Δ::*URA3-P*_*STE18*_*-Gγ*_*cyto*_*-Z*_*WT*_	This study
UG2-FCGK	BYFUG2 *ste18*Δ::*kanMX4-P*_*PGK1*_*-Fc-Ste18C his3*Δ::*URA3-P*_*STE18*_*-Gγ*_*cyto*_*-Z*_*K35A*_	This study
UG2-FCGI	BYFUG2 *ste18*Δ::*kanMX4-P*_*PGK1*_*-Fc-Ste18C his3*Δ::*URA3-P*_*STE18*_*-Gγ*_*cyto*_*-Z*_*I31A*_	This study
UG2-FCG9	BYFUG2 *ste18*Δ::*kanMX4-P*_*PGK1*_*-Fc-Ste18C his3*Δ::*URA3-P*_*STE18*_*-Gγ*_*cyto*_*-Z*_*955*_	This study
UG2-FCG0	BYFUG2 *ste18*Δ::*kanMX4-P*_*PGK1*_*-Fc-Ste18C his3*Δ::*URA3*	This study
UG2-FNGW	BYFUG2 *ste18*Δ::*kanMX4-P*_*PGK1*_*-Gpa1N-Fc his3*Δ::*URA3-P*_*STE18*_*-Gγ*_*cyto*_*-Z*_*WT*_	This study
UG2-FNGK	BYFUG2 *ste18*Δ::*kanMX4-P*_*PGK1*_*-Gpa1N-Fc his3*Δ::*URA3-P*_*STE18*_*-Gγ*_*cyto*_*-Z*_*K35A*_	This study
UG2-FNGI	BYFUG2 *ste18*Δ::*kanMX4-P*_*PGK1*_*-Gpa1N-Fc his3*Δ::*URA3-P*_*STE18*_*-Gγ*_*cyto*_*-Z*_*I31A*_	This study
UG2-FNG9	BYFUG2 *ste18*Δ::*kanMX4-P*_*PGK1*_*-Gpa1N-Fc his3*Δ::*URA3-P*_*STE18*_*-Gγ*_*cyto*_*-Z*_*955*_	This study
UG2-FNG0	BYFUG2 *ste18*Δ::*kanMX4-P*_*PGK1*_*-Gpa1N-Fc his3*Δ::*URA3*	This study

**Table 4:** Codon adaptation index (CAI) and GC content of varied GFPs tested.

Protein	CAI	GC content (%)
EGFP	0.52	61.8
AcGFP1	0.56	59.0
TagGFP2	0.50	63.6
mUkG1	0.69	45.0
ZsGreen	0.70	44.9
yEGFP	0.87	40.4
yAcGFP1	0.87	40.2
yTagGFP2	0.87	40.2
ymUkG1	0.88	40.1
yZsGreen	0.90	40.0
ymWasabi	0.87	39.7
ymNeonGreen	0.90	40.0

**Table 5:** Summary of FACS analysis of cell mixtures containing positive and negative cells.

Reporter	Interaction target	Initial ratio of target cells in mixture	Rate of detected target cell number against initial target cell number
EGFP	Z_WT_	0.1%	33%
EGFP	Z_WT_	0.01%	34%
ymUkG1	Z_WT_	0.1%	58%
ymUkG1	Z_WT_	0.01%	56%
EGFP	Z_I31A_	0.1%	12%
EGFP	Z_I31A_	0.01%	24%
ymUkG1	Z_I31A_	0.1%	74%
ymUkG1	Z_I31A_	0.01%	65%
