# Supplementary material for: Expression of varied GFPs in Saccharomyces cerevisiae: codon optimization yields stronger than expected expression and fluorescence intensity
Source: Sci Rep. 2016 Oct 26;6:35932. doi: 10.1038/srep35932 (PMC5080575; doi:10.1038/srep35932)
Supplement: Supplementary Information [file srep35932-s1.pdf]

Expression of varied GFPs in *Saccharomyces cerevisiae*: codon optimization yields stronger than expected expression and fluorescence intensity

Misato Kaishima[1], Jun Ishii[2], Toshihide Matsuno[2], Nobuo Fukuda[3] and Akihiko Kondo[1]

[1]Department of Chemical Science and Engineering, Graduate School of Engineering, Kobe University, 1-1 Rokkodai, Nada, Kobe 657-8501, Japan
[2]Graduate School of Science, Technology and Innovation, Kobe University, 1-1 Rokkodai, Nada, Kobe 657-8501, Japan
[3]Biomedical Research Institute, National Institute of Advanced Industrial Science and Technology (AIST), Higashi, Tsukuba, Japan

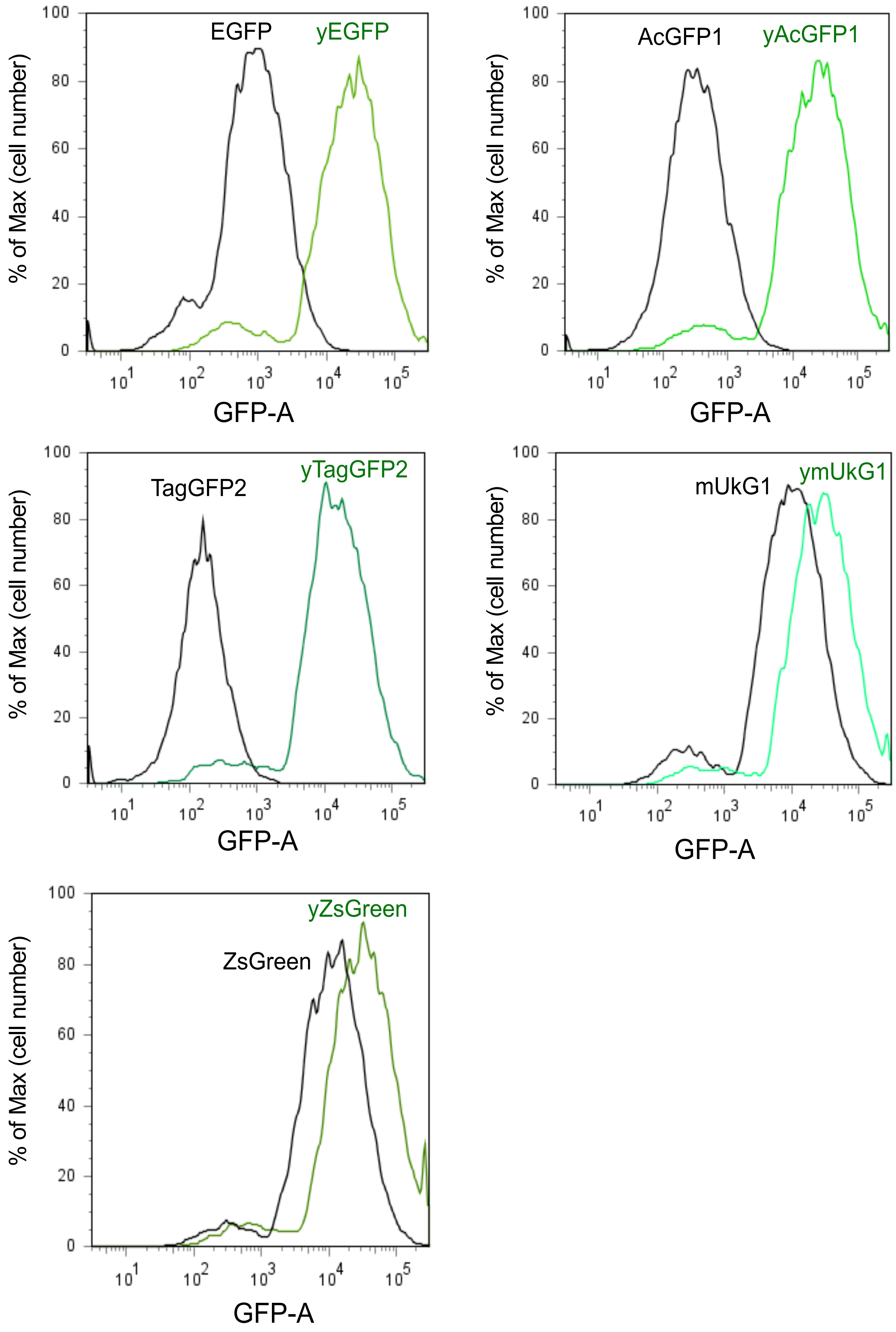

Figure S1. Histogram plots illustrating codon-optimized and non-codon-optimized GFPs expression in *S. cerevisiae.* The histogram plots show analytical data measured for 10,000 cells using flow cytometry.

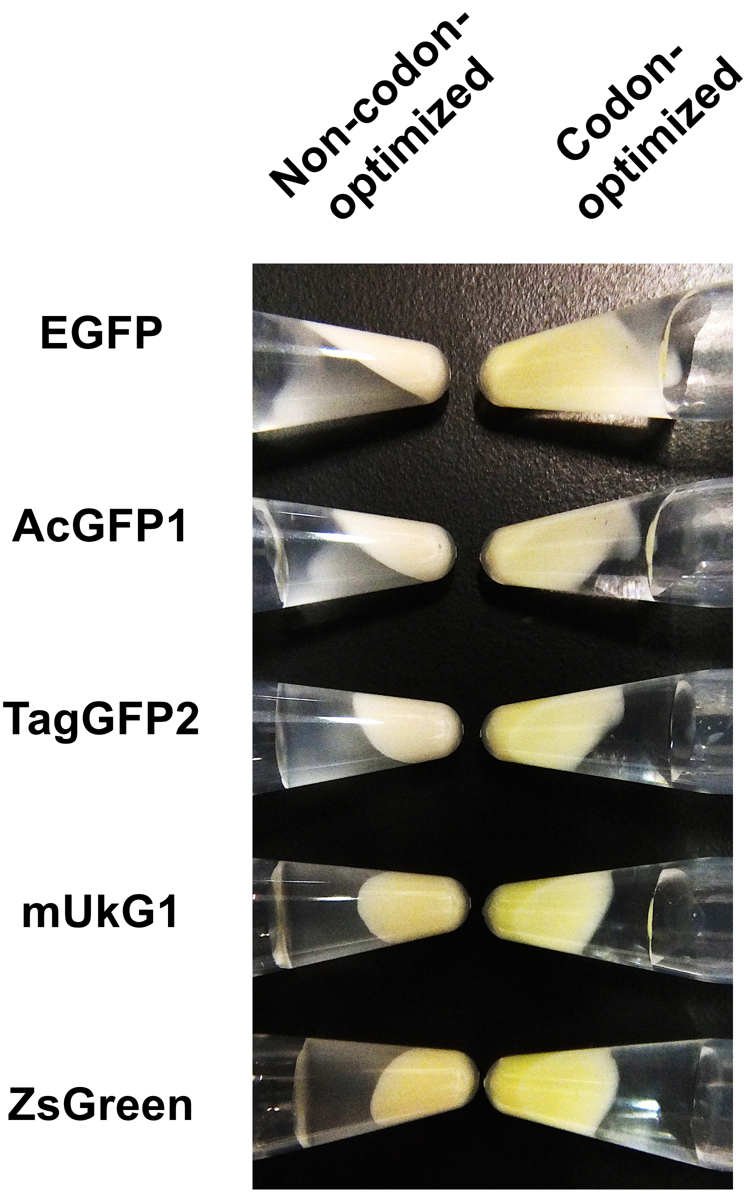

Figure S2. Visual images of the cells under natural light. Varied codon-optimized and non-codon-optimized GFPs were used in the evaluations.

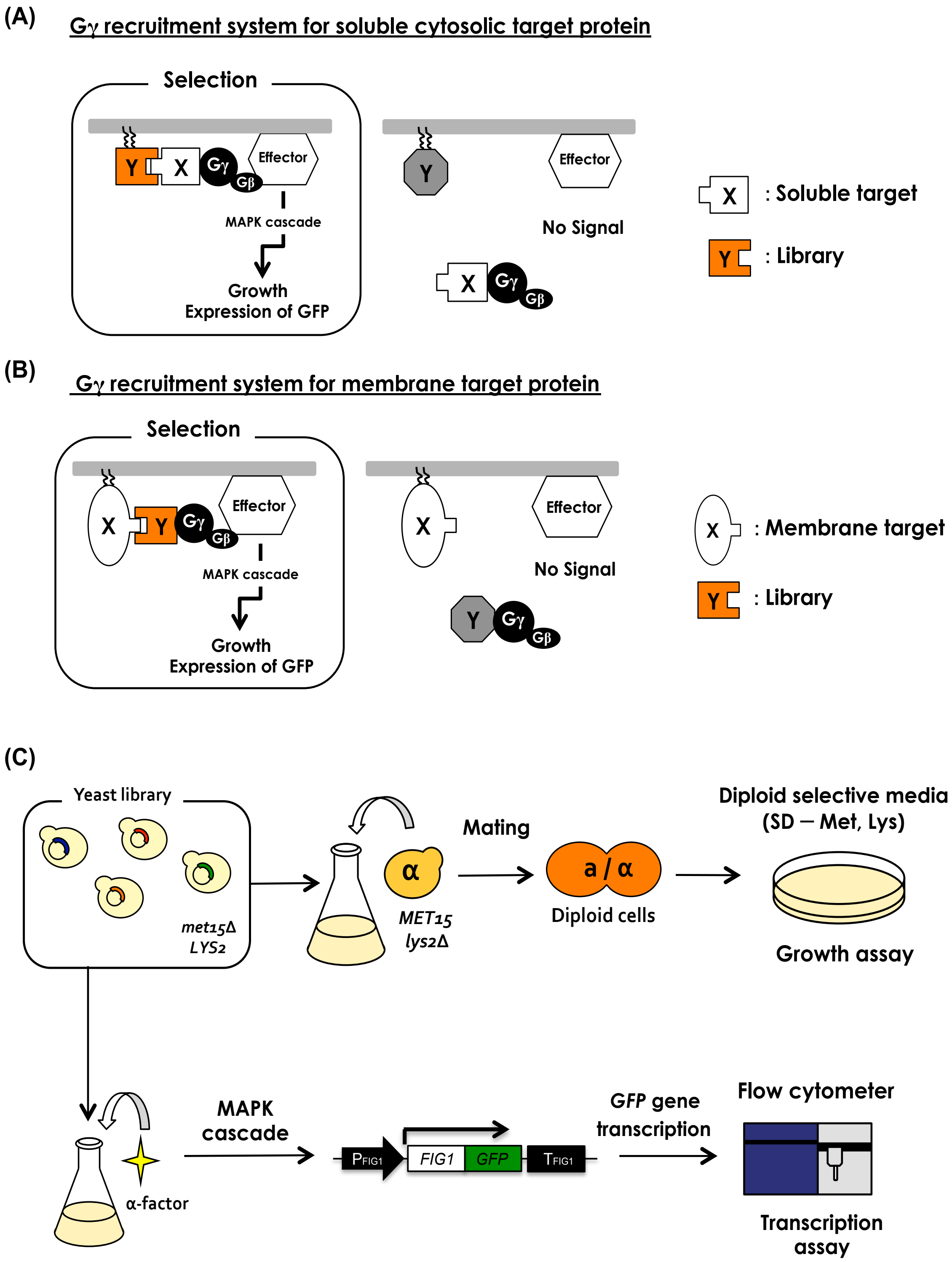

Figure S3. Schematic diagram of Gγ recruitment systems used to detect PPIs of cytosolic or membrane target proteins. (A) Schematic outline of the Gγ recruitment system for cytosolic target proteins. When target protein 'X', which is fused to $G\gamma_{cyto}$, interacts with candidate protein 'Y_1,' the Gβ and $G\gamma_{cyto}$ complex ($G\beta\gamma_{cyto}$) migrates to the inner leaflet of the plasma membrane and restores the signaling function. If protein 'X' cannot interact with protein 'Y' $G\beta\gamma_{cyto}$ is released into the cytosol, and signaling is blocked. (B) Schematic outline of the Gγ recruitment system for membrane protein targets. When membrane target protein 'X' interacts with candidate protein 'Y', which is fused to $G\gamma_{cyto}$, the $G\beta\gamma_{cyto}$ complex migrates to the inner leaflet of the plasma membrane and restores the signaling function. If membrane protein 'X' cannot interact with protein 'Y,' $G\beta\gamma_{cyto}$ is released into the cytosol, and signaling is blocked. (C) Flow diagram of the screening procedure used in the Gγ recruitment system. Two selection methods are available to screen for new binding proteins. One method uses *GFP* reporter genes. When target candidate proteins are expressed in yeast cells and interact with each other, the proteins induce *GFP* expression, which is detected by flow cytometry.

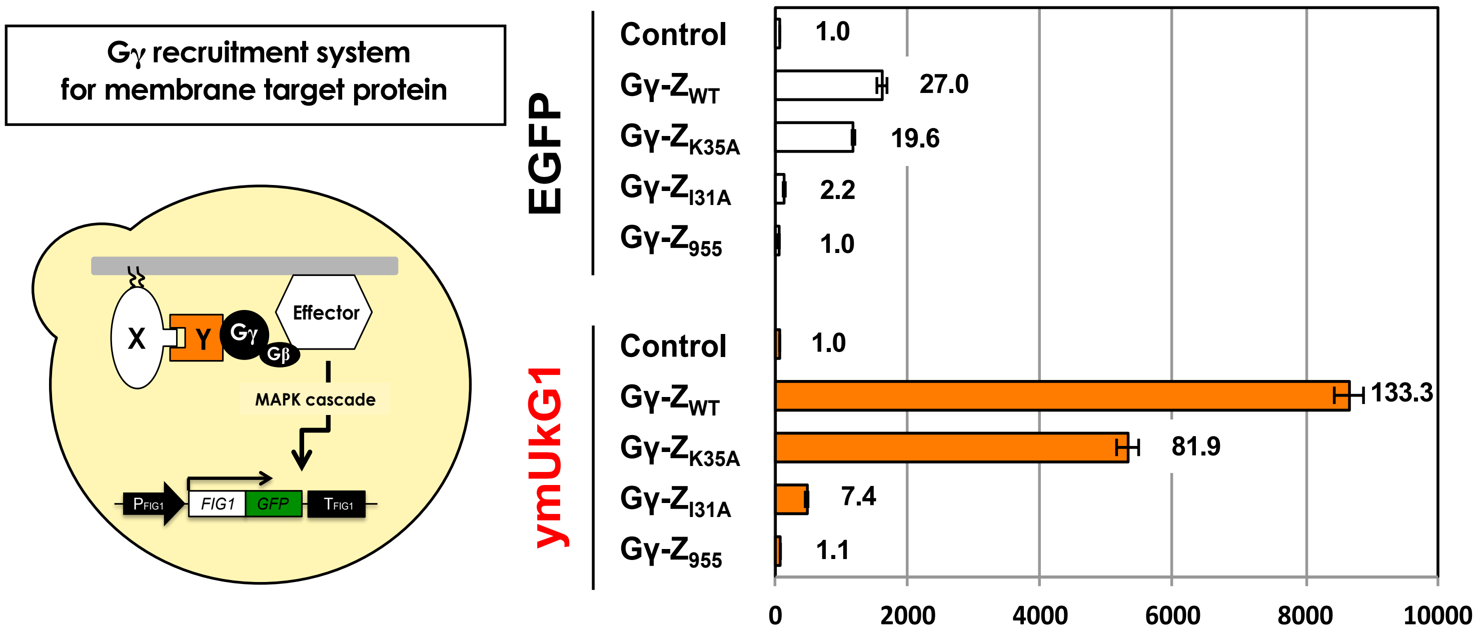

Figure S4. Expression of non-codon-optimized EGFP and codon-optimized ymUkG1 as fusion-tagged proteins to detect PPIs using the Gγ recruitment system.
Flow cytometry analyses using the Gγ recruitment system for membrane protein targets. The Fc protein was used as the membrane target protein 'X' and was expressed as a membrane-associated protein with an N-terminal lipid anchor (derived from Gpa1p). Four Z variants (Z_{WT}, $Z_{K35A,}$, Z_{I31A} and Z_{955}) were used as the cytosolic candidate 'Y' proteins and were expressed as fusion proteins with $G\gamma_{cyto}$ ($G\gamma_{cyto}$-Z variants). 'Control' indicates FN-G0 and UG2-FNG0 yeast strains without the expression of '$G\gamma_{cyto}$-Y'. The engineered strains were grown in media containing 5 μM α-factor and were used in the analyses of mean fluorescence intensities (MFIs). The MFIs of 10,000 cells were measured by flow cytometry.

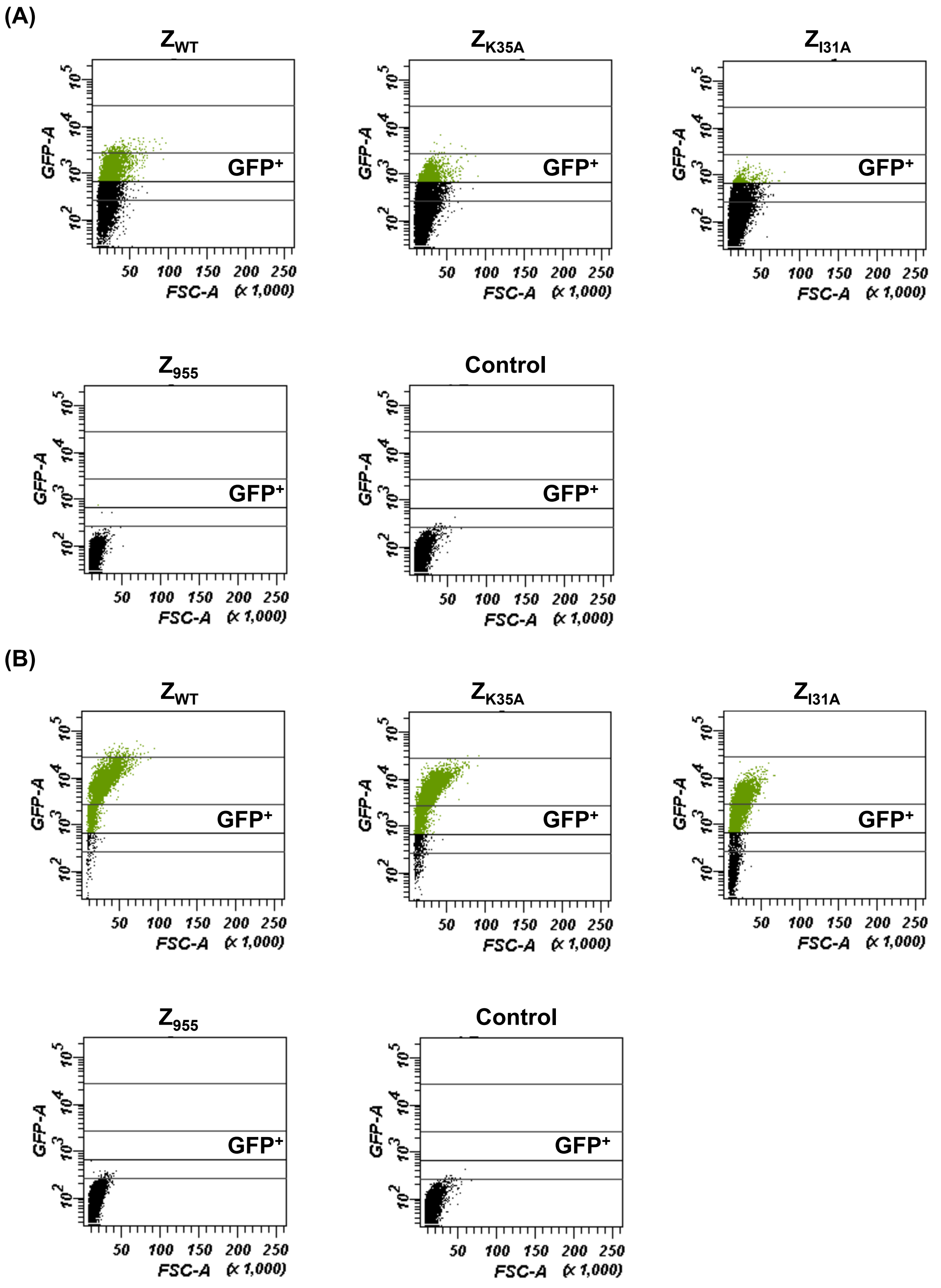

Figure S5. Determination of gate area to dominantly including positive fluorescent cells (GFP⁺). Data were presented as dot plots (forward scatter, FSC-A vs green fluorescence, GFP). Y-axis is an indication of fluorescence and X-axis is an approximation of relative cell size. Positive cells express membrane-anchored Z_{WT}, Z_{K35A} and Z_{I31A} with $G\gamma_{cyto}$-Fc. Negative cells express membrane-anchored Z_{955} with $G\gamma_{cyto}$-Fc. 'Control' indicates BFG2118 and UGFG2 yeast strains harboring the pGK413 mock plasmid (without the expression of 'Y'). (A) Flow cytometry analysis of the BFG2118 yeast transformants (EGFP reporter). (B) Flow cytometry analysis of the UGFG2 yeast transformants (ymUkG1 reporter).

Supplementary Table S1. Nucleic acid sequences of codon-optimized GFPs for the *Saccharomyces cerevisiae*

yEGFP

ATGGTCAGTAAGGGTGAAGAATTATTCACTGGTGTTGTTCCAATCTTGGTTGAATTGGATGGTGATGTTAACGGTCAC
AAGTTTTCTGTTTCTGGTGAAGGTGAAGGTGATGCTACTTATGGTAAATTGACCTTGAAGTTCATCTGTACCACAGGT
AAATTGCCAGTTCCATGGCCAACTTTGGTTACTACTTTGACTTATGGTGTCCAATGCTTCTCTAGATACCCAGATCATA
TGAAGCAACACGACTTTTTCAAATCCGCTATGCCAGAAGGTTACGTTCAAGAAAGAACCATCTTCTTCAAGGATGAC
GGTAACTACAAAACTAGAGCCGAAGTTAAGTTCGAAGGTGATACCTTGGTTAACAGAATCGAATTGAAGGGTATCGAC
TTCAAAGAAGATGGTAACATCTTGGGTCATAAGTTGGAATACAACTACAACTCCCACAACGTTTACATTATGGCCGATA
AGCAAAAGAACGGTATCAAGGTTAACTTCAAGATCAGACACAACATCGAAGATGGTAGTGTTCAATTGGCTGATCACT
ACCAACAAAACACTCCAATTGGTGATGGTCCAGTTTTGTTGCCAGATAACCATTACTTGTCTACCCAATCTGCTTTGT
CTAAGGACCCAAACGAAAAAAGAGATCACATGGTCTTGTTGGAATTCGTTACTGCTGCTGGTATTACTTTGGGTATGG
ACGAATTATACAAGTAA

yAcGFP1

ATGGTTTCTAAGGGTGCTGAATTATTCACTGGTATCGTTCCAATCTTGATCGAATTGAACGGTGATGTTAACGGTCACA
AGTTTTCTGTTTCTGGTGAAGGTGAAGGTGATGCTACTTATGGTAAATTGACCTTGAAGTTCATCTGTACCACAGGTA
AATTGCCAGTTCCATGGCCAACTTTGGTTACTACTTTGTCTTATGGTGTCCAATGCTTCTCTAGATACCCAGATCATAT
GAAGCAACACGACTTTTTCAAATCCGCTATGCCAGAAGGTTACATCCAAGAAAGAACCATCTTTTTCGAAGATGACG
GTAACTACAAGTCTAGAGCTGAAGTTAAGTTCGAAGGTGATACCTTGGTTAACAGAATTGAATTGACCGGTACTGACT
TCAAAGAAGATGGTAACATTTTGGGTAACAAGATGGAATACAACTACAACGCCCATAACGTTTACATCATGACTGATAA
GGCTAAGAACGGTATCAAGGTTAACTTCAAGATCAGACACAACATCGAAGATGGTTCAGTTCAATTGGCTGATCACTA
CCAACAAAACACTCCAATTGGTGATGGTCCAGTTTTGTTGCCAGATAACCATTACTTGTCTACCCAATCTGCTTTGTCT
AAGGACCCAAACGAAAAAAGAGATCACATGATCTACTTCGGTTTCGTTACTGCTGCTGCTATTACTCATGGTATGGAC
GAATTATACAAGTAA

yTagGFP2

ATGTCTGGTGGTGAAGAATTATTCGCTGGTATAGTTCCAGTCTTGATCGAATTGGATGGTGATGTTCATGGTCACAAG
TTTTCTGTTAGAGGTGAAGGTGAAGGTGATGCTGATTATGGTAAATTGGAAATCAAGTTCATCTGCACCACAGGTAAA
TTGCCAGTTCCATGGCCAACTTTGGTTACTACTTTGTGTTACGGTATTCAATGCTTCGCTAGATACCCAGAACATATGA
AGATGAACGACTTCTTCAAATCCGCTATGCCAGAAGGTTACATTCAAGAAAGAACCATTCAATTCCAAGACGACGGTA
AATACAAAACCAGAGGTGAAGTTAAGTTCGAAGGTGATACTTTGGTCAACAGAATCGAATTGAAGGGTAAGGACTTC

AAAGAAGATGGTAACATCTTGGGTCATAAGTTGGAATACTCATTCAACTCCCACAACGTTTACATCAGACCAGATAAG

GCTAACAATGGTTTGGAAGCTAACTTCAAGACCAGACATAATATTGAAGGTGGTGGTGTTCAATTGGCCGATCATTAT

CAAACTAATGTTCCATTGGGTGACGGTCCAGTTTTGATTCCAATCAATCATTACTTGTCCACCCAAACTAAGATCTCCA

AGGATAGAAATGAAGCCAGAGATCACATGGTCTTGTTGGAATCTTTTTCTGCTTGTTGTCATACCCACGGTATGGACG

AATTATACAGATGA

ymUkG1

ATGGTCAGTGTCATCAAAGAAGAAATGAAGATCAAGTTGCACATGGAAGGTAACGTTAATGGTCATGCCTTTGTTATT

GAAGGTGATGGTAAAGGTAAACCATACGATGGTACTCAAACTTTGAACTTGACTGTCAAAGAAGGTGCTCCATTGCC

ATTCTCTTACGATATTTTGACTAACGCCTTCCAATACGGTAATAGAGCTTTTACTAAGTACCCAGCCGATATCCCAGATT

ACTTTAAGCAAACTTTTCCAGAAGGTTACTCCTGGGAAAGAACTATGTCTTACGAAGATAACGCTATCTGCAACGTCA

GATCCGAAATTTCTATGGAAGGTGATTGCTTCATCTACAAGATCAGATTCGATGGTAAGAACTTTCCACCAAATGGTC

CAGTCATGCAAAAAAAGACTTTGAAGTGGGAACCATCCACCGAAATGATGTATGTTAGAGATGGTTTCTTGATGGGT

GATGTCAATATGGCTTTGTTGTTGGAAGGTGGTGGTCATCATAGATGTGATTTCAAGACTTCTTACAAGGCCAAGAAG

GTTGTTCAATTGCCAGATGCTCATAAGATCGATCACAGAATCGAAATCTTGTCCCACGATAGAGATTACTCCAAGGTTA

AGTTGTACGAAAACGCTGTTGCTAGAAACTCTTTGTTGCCATCTCAAGCTTCTAAGTAA

yZsGreen

ATGGCTCAATCCAAACATGGTTTGACCAAAGAAATGACCATGAAGTACAGAATGGAAGGTTGTGTTGATGGTCACAA

GTTCGTTATTACTGGTGAAGGTATTGGTTACCCATTCAAAGGTAAGCAAGCTATCAACTTGTGTGTTGTTGAAGGTGG

TCCATTGCCATTTGCTGAAGATATTTTGTCTGCTGCTTTCATGTACGGTAACAGAGTTTTTACTGAATACCCACAAGAT

ATCGCCGACTACTTTAAGAATTCATGTCCAGCTGGTTACACCTGGGATAGATCTTTTTTGTTTGAAGATGGTGCTGTC

TGTATCTGCAACGCTGATATTACTGTTTCCGTTGAAGAAAACTGCATGTACCACGAATCTAAGTTCTACGGTGTTAATT

TTCCAGCTGATGGTCCAGTTATGAAGAAGATGACTGATAATTGGGAACCATCCTGCGAAAAGATTATTCCAGTTCCAA

AGCAAGGTATCTTGAAGGGTGATGTTTCTATGTACTTGTTATTGAAGGACGGTGGTAGATTGAGATGTCAATTCGATA

CTGTTTACAAGGCCAAGTCTGTTCCAAGAAAAATGCCTGATTGGCATTTCATCCAACATAAGTTGACCAGAGAAGATA

GATCCGATGCCAAAAATCAAAAGTGGCATTTGACCGAACATGCTATTGCTTCAGGTTCTGCTTTGCCATAA

ymWasabi

ATGGTCAGTAAGGGTGAAGAAACTACTATGGGTGTTATCAAGCCAGACATGAAGATCAAGTTGAAGATGGAAGGTAA

CGTTAACGGTCATGCCTTTGTTATTGAAGGTGAAGGTGAAGGTAAACCATACGATGGTACTAATACCATTAACTTGGA

AGTCAAAGAAGGTGCTCCATTGCCATTCTCTTACGATATTTTGACTACCGCTTTCTCATACGGTAATAGAGCTTTTACT

AAGTACCCAGATGACATCCCAAACTACTTCAAGCAATCTTTTCCAGAAGGTTACTCTTGGGAAAGAACTATGACTTTC

GAAGATAAGGGTATCGTCAAGGTTAAGTCCGATATCTCTATGGAAGAAGATTCCTTCATCTACGAAATCCACTTGAAG

GGTGAAAATTTCCCACCAAATGGTCCAGTCATGCAAAAAGAAACAACTGGTTGGGATGCTTCTACCGAAAGAATGTA

TGTTAGAGATGGTGTCTTGAAAGGTGACGTCAAAATGAAGTTGTTGTTGGAAGGTGGTGGTCATCATAGAGTTGATT

TCAAGACTATCTACAGAGCTAAGAAGGCTGTTAAGTTGCCAGATTACCATTTCGTTGATCACAGAATCGAAATCTTGA

ACCACGATAAGGATTACAACAAGGTTACCGTTTACGAAATTGCTGTTGCTAGAAACTCTACCGATGGTATGGATGAATT

ATACAAGTAA

ymNeonGreen

ATGGTCAGTAAGGGTGAAGAAGATAACATGGCTTCTTTGCCAGCTACTCATGAATTGCATATTTTCGGTTCCATCAAC

GGTGTTGATTTCGATATGGTTGGTCAAGGTACTGGTAATCCAAATGATGGTTACGAAGAATTGAACTTGAAGTCTACTA

AGGGTGACTTGCAATTCTCTCCATGGATTTTGGTTCCACATATTGGTTACGGTTTCCACCAATATTTGCCATATCCAGA

TGGTATGTCTCCATTTCAAGCTGCTATGGTTGATGGTTCTGGTTACCAAGTTCATAGAACCATGCAATTTGAAGATGGT

GCTTCTTTGACCGTCAACTACAGATATACTTACGAAGGTTCCCATATCAAAGGTGAAGCTCAAGTTAAGGGTACTGGT

TTTCCAGCTGATGGTCCAGTTATGACTAATTCTTTGACTGCTGCTGATTGGTGCAGATCTAAAAAGACTTACCCAAAC

GATAAGACCATCATCTCTACTTTCAAGTGGTCTTACACTACTGGTAACGGTAAGAGATATAGATCTACTGCTAGAACTA

CTTACACCTTCGCTAAACCTATGGCTGCTAACTACTTGAAGAATCAACCTATGTACGTTTTCAGAAAGACCGAATTGAA

GCACTCCAAAACTGAATTGAATTTCAAAGAATGGCAAAAGGCCTTCACCGATGTTATGGGTATGGATGAATTATACAA

GTAA

Supplementary Table S2. List of yeast transformants used for expression of various GFPs

Transformant	Expression of protein	Figure
BY4741 (Control)	–	Fig.1
BY4741 + pGK416-EGFP	EGFP (mammalian codon-optimized)	Figs.1 and S2
BY4741 + pGK416-AcGFP1	AcGFP1 (mammalian codon-optimized)	Figs.1 and S2
BY4741 + pGK416-TagGFP2	TagGFP2 (mammalian codon-optimized)	Figs.1 and S2
BY4741 + pGK416-mUkG1	mUkG1	Figs.1 and S2
BY4741 + pGK416-ZsGreen	ZsGreen	Figs.1 and S2
BY4741 + pGK416-yEGFP	yEGFP (yeast codon-optimized)	Figs.1 , S1 and S2
BY4741 + pGK416-yAcGFP1	yAcGFP1 (yeast codon-optimized)	Figs.1 , S1 and S2
BY4741 + pGK416-yTagGFP2	yTagGFP2 (yeast codon-optimized)	Figs.1 , S1 and S2
BY4741 + pGK416-ymUkG1	ymUkG1 (yeast codon-optimized)	Figs.1 , S1 and S2
BY4741 + pGK416-yZsGreen	yZsGreen (yeast codon-optimized)	Figs.1 , S1 and S2
BY4741 + pGK416-ymWasabi	ymWasabi (yeast codon-optimized)	Fig. S1
BY4741 + pGK416-ymNeonGreen	ymNeonGeen (yeast codon-optimized)	Fig. S1
BY4741 + pGK416-EGFP-F	EGFP (mammalian codon-optimized)-FLAG	Fig.1
BY4741 + pGK416-AcGFP1-F	AcGFP1 (mammalian codon-optimized)-FLAG	Fig.1
BY4741 + pGK416-TagGFP2-F	TagGFP2 (mammalian codon-optimized)-FLAG	Fig.1
BY4741 + pGK416-mUkG1-F	mUkG1-FLAG	Fig.1
BY4741 + pGK416-ZsGreen-F	ZsGreen-FLAG	Fig.1
BY4741 + pGK416-yEGFP-F	yEGFP (yeast codon-optimized)-FLAG	Fig.1
BY4741 + pGK416-yAcGFP1-F	yAcGFP1 (yeast codon-optimized)-FLAG	Fig.1
BY4741 + pGK416-yTagGFP2-F	yTagGFP2 (yeast codon-optimized)-FLAG	Fig.1
BY4741 + pGK416-ymUkG1-F	ymUkG1 (yeast codon-optimized)-FLAG	Fig.1
BY4741 + pGK416-yZsGreen-F	yZsGreen (yeast codon-optimized)-FLAG	Fig.1
BY4741 + pGK416-ymWasabi-F	ymWasabi (yeast codon-optimized)-FLAG	Fig.1
BY4741 + pGK416-ymNeonGreen-F	ymNeonGeen (yeast codon-optimized)-FLAG	Fig.1

Supplementary Table S3. Mean fluorescence intensity (MFI) of yeast transformants used for expression of various GFPs

GFP	MFI
Control	36
EGFP	1,490
AcGFP1	542
TagGFP2	240
mUkG1	14,194
ZsGreen	15,019
yEGFP	33,551
yAcGFP1	36,177
yTagGFP2	24,250
ymUkG1	47,088
yZsGreen	44,154
ymWasabi	16,987
ymNeonGreen	5,148

Supplementary Table S4. List of yeast strains used for expression of various Fig1-GFPs as fusion tagged reporters

Strain	Expression of protein	Figure
BY4741 (Control)	–	Fig. 2
MC-F1	Fig1-EGFP (mammalian codon-optimized)	Fig. 2
BYFAG1	Fig1-AcGFP1 (mammalian codon-optimized)	Fig. 2
BYFTG1	Fig1-TagGFP2 (mammalian codon-optimized)	Fig. 2
BYFUG1	Fig1-mUkG1	Fig. 2
BYFZG1	Fig1-ZsGreen	Fig. 2
BYFEG2	Fig1-yEGFP (yeast codon-optimized)	Fig. 2
BYFAG2	Fig1-yAcGFP1 (yeast codon-optimized)	Fig. 2
BYFTG2	Fig1-yTagGFP2 (yeast codon-optimized)	Fig. 2
BYFUG2	Fig1-ymUkG1 (yeast codon-optimized)	Fig. 2
BYFZG2	Fig1-yZsGreen (yeast codon-optimized)	Fig. 2

Supplementary Table S5. List of yeast transformants used for the Gγ recruitment system for soluble cytosolic target protein

Transformant	Target<br>X (cytosol)	Candidate<br>Y (membrane)	Repoter<br>GFP	Figure
BFG2118 + pGk413 (Control)	Fc	–	EGFP	Figs. 3(A) and S5(A)
BFG2118 + pGK413-ZWTmem	Fc	Z_{WT}	EGFP	Figs. 3(A), 4(A), 4(B) and S5(A)
BFG2118 + pGK413-ZK35Amem	Fc	Z_{K35A}	EGFP	Figs. 3(A) and S5(A)
BFG2118 + pGK413-ZI31Amem	Fc	Z_{I31A}	EGFP	Figs. 3(A), 4(E), 4(F) and S5(A)
BFG2118 + pGK413-Z955mem	Fc	Z_{955}	EGFP	Figs. 3(A), 4(A), 4(B), 4(E), 4(F) and S5(A)
UGFG2 + pGk413 (Control)	Fc	–	ymUkG1	Figs. 3(A) and S5(B)
UGFG2 + pGK413-ZWTmem	Fc	Z_{WT}	ymUkG1	Figs. 3(A), 4(C), 4(D) and S5(B)
UGFG2 + pGK413-ZK35Amem	Fc	Z_{K35A}	ymUkG1	Figs. 3(A) and S5(B)
UGFG2 + pGK413-ZI31Amem	Fc	Z_{I31A}	ymUkG1	Figs. 3(A), 4(G), 4(H) and S5(B)
UGFG2 + pGK413-Z955mem	Fc	Z_{955}	ymUkG1	Figs. 3(A), 4(C), 4(D), 4(G), 4(H) and S5(B)

Supplementary Table S6. List of yeast strains used for the Gγ recruitment system for membrane target protein

Strain	Target<br>X (membrane)	Candidate<br>Y (cytosol)	Repoter GFP	Figure
FC-G0 (Control)	C-terminally membrane-associated Fc	–	EGFP	Fig. 3(B)
FC-GW	C-terminally membrane-associated Fc	Z_{WT}	EGFP	Fig. 3(B)
FC-GK	C-terminally membrane-associated Fc	Z_{K35A}	EGFP	Fig. 3(B)
FC-GI	C-terminally membrane-associated Fc	Z_{I31A}	EGFP	Fig. 3(B)
FC-G9	C-terminally membrane-associated Fc	Z_{955}	EGFP	Fig. 3(B)
FN-G0 (Control)	N-terminally membrane-associated Fc	–	EGFP	Fig. S4
FN-GW	N-terminally membrane-associated Fc	Z_{WT}	EGFP	Fig. S4
FN-GK	N-terminally membrane-associated Fc	Z_{K35A}	EGFP	Fig. S4
FN-GI	N-terminally membrane-associated Fc	Z_{I31A}	EGFP	Fig. S4
FN-G9	N-terminally membrane-associated Fc	Z_{955}	EGFP	Fig. S4
UG2-FCG0	C-terminally membrane-associated Fc	–	ymUkG1	Fig. 3(B)
UG2-FCGW	C-terminally membrane-associated Fc	Z_{WT}	ymUkG1	Fig. 3(B)
UG2-FCGK	C-terminally membrane-associated Fc	Z_{K35A}	ymUkG1	Fig. 3(B)
UG2-FCGI	C-terminally membrane-associated Fc	Z_{I31A}	ymUkG1	Fig. 3(B)
UG2-FCG9	C-terminally membrane-associated Fc	Z_{955}	ymUkG1	Fig. 3(B)
UG2-FNG0	N-terminally membrane-associated Fc	–	ymUkG1	Fig. S4
UG2-FNGW	N-terminally membrane-associated Fc	Z_{WT}	ymUkG1	Fig. S4
UG2-FNGK	N-terminally membrane-associated Fc	Z_{K35A}	ymUkG1	Fig. S4
UG2-FNGI	N-terminally membrane-associated Fc	Z_{I31A}	ymUkG1	Fig. S4
UG2-FNG9	N-terminally membrane-associated Fc	Z_{955}	ymUkG1	Fig. S4

Supplementary Table S7. Summary of the FACS analysis of the Gγ recruitment system for cytosolic target proteins. The percentage of cells belonging to GFP^+ were measured by flow cytometry.

Reporter GFP	Type of Z variant	Percentage of GFP^+
EGFP	Z_{WT}	58.8
EGFP	Z_{K35A}	13.9
EGFP	Z_{I31A}	2.5
EGFP	Z_{955}	0
EGFP	Control	0
ymUkG1	Z_{WT}	98.3
ymUkG1	Z_{K35A}	95.4
ymUkG1	Z_{I31A}	80.3
ymUkG1	Z_{955}	0
ymUkG1	Control	0

Supplementary Table S8. List of primers

No.	Name	Sequence (5' to 3)
1	SalI-start-AcGFP1-fw	aaaagtcgacatggtgagcaagggc
2	BamHI-end-AcGFP1-rv	ttttggatcctcacttgtacagctcat
3	SalI-start-TagGFP2-fw	aaaagtcgacatgagcgggggcgaggag
4	BamHI-end-TagGFP2-rv	ttttggatccttacctgtacagctcgtc
5	SalI-start-mUkG1-fw	aaaagtcgacatggtgagtgtgattaaa
6	BamHI-end-mUkG1-rv	ttttggatccttacttcgaagcctgact
7	SalI-start-EGFP(Yeast)-fw	aaaagtcgacatggtcagtaagggtgaa
8	BamHI-end-EGFP(Yeast)-rv	ttttggatccttacttgtataattcgtc
9	SalI-start-AcGFP1(Yeast)-fw	aaaagtcgacatggtttctaagggtgct
10	BamHI-end-AcGFP1(Yeast)-rv	ttttggatccttacttgtataattcgtc
11	SalI-start-TagGFP2(Yeast)-fw	aaaagtcgacatgtctggtggtgaagaa
12	BamHI-end-TagGFP2(Yeast)-rv	ttttggatcctcatctgtataattcgtc
13	SalI-start-mUkG1(Yeast)-fw	aaaagtcgacatggtcagtgtcatcaaaga
14	BamHI-end-mUkG1(Yeast)-rv	ttttggatccttacttagaagcttgagatg
15	SalI-start-ZsGreen(Yeast)-fw	aaaagtcgacatggctcaatccaaacat
16	BamHI-end-ZsGreen (Yeast)-rv	ttttggatccttatggcaaagcagaacc
17	SalI-start-mWasabi (Yeast)-fw	tagcgtcgacatggtgagcaagggcgagg
18	BamHI-end-mWasabi (Yeast)-rv	tagacccgggttacttgtataattcatccataccatcgg
19	SalI-start-mNeonGreen(Yeast)-fw	agcgtcgacatggtcagtaagggtgaaga
20	BamHI-end-mNeonGreen (Yeast)-rv	cgggggatccttacttgtataattcatc
21	SalI-atg-EGFP-fw	tagcgtcgacatggtgagcaagggcgagga
22	BamHI-end-frag-EGFP-rv	cgggggatccttacttgtcatcgtcatccttgtagtccttgtacagctcgtccatgccga
23	BamHI-end-frag-AcGFP1-rv	cgggggatcctcacttgtcatcgtcatccttgtagtccttgtacagctcatccatgccgt
24	BamHI-end-frag-TagGFP2-rv	cgggggatccttacttgtcatcgtcatccttgtagtccctgtacagctcgtccatgccgt
25	BamHI-end-frag-mUkG1-rv	cgggggatccttacttgtcatcgtcatccttgtagtccttcgaagcctgacttggcagca
26	SalI-atg-ZsGreen-fw	tagcgtcgacatggctcagtcaaagcac
27	BamHI-end-frag-ZsGreen-rv	cgggggatcctcacttgtcatcgtcatccttgtagtcgggcaatgcagatccggatgcaa
28	BamHI-end-frag-yEGFP-rv	cgggggatccttacttgtcatcgtcatccttgtagtccttgtataattcgtccataccca
29	BamHI-end-frag-yAcGFP1-rv	cgggggatccttacttgtcatcgtcatccttgtagtccttgtataattcgtccataccat

30	BamHI-end-frag-yTagGFP2-rv	cgggggatcctcacttgtcatcgtcatccttgtagtctctgtataattcgtccataccgt
31	BamHI-end-frag-ymUkG1-rv	cgggggatccttacttgtcatcgtcatccttgtagtccttagaagcttgagatggcaaca
32	BamHI-end-frag-yZsGreen-rv	cgggggatccttacttgtcatcgtcatccttgtagtctggcaaagcagaacctgaagcaa
33	BamHI-end-frag-ymWasabi-rv	cgggggatccttacttgtcatcgtcatccttgtagtccttgtataattcatccataccat
34	BamHI-end-frag-ymNeonGreen-rv	cgggggatccttacttgtcatcgtcatccttgtagtccttgtataattcatccataccca
35	XhoI-FIG1down-fw	ggggctcgagttttatcctcaaataaacat
36	KpnI-FIG1down-rv	ccccggtaccaacagacggtaatgattaga
37	TFIG1hr40-URA3-fw	ttttatcctcaaataaacatataagttttgagcggatatttttttgttcttttttttgattccg
38	XhoI-URA3-rv	gaggataaaactcgaggggtaataactgatataatt
39	SacII-FIG1end50-AcGFP1-fw	aaaaccgcggataggtacaataactactcttcggattcatctacattgcattccaaagttgtgagca agggcgccgagctgttcaccggc
40	AcGFP1-TFIG1hr40-rv	aatatccgctcaaaacttatatgtttatttgaggataaaatcacttgtacagctcatccatgccgtgg gt
41	SacII-FIG1end50-TagGFP2-fw	aaaaccgcggataggtacaataactactcttcggattcatctacattgcattccaaagttagcggg ggcgaggagctgttcgccggc
42	TagGFP2-TFIG1hr40-rv	aatatccgctcaaaacttatatgtttatttgaggataaaattacctgtacagctcgtccatgccg
43	SacII-FIG1end50-mUkG1-fw	aaaaccgcggataggtacaataactactcttcggattcatctacattgcattccaaagttgtgagtg tgattaaagagga
44	mUkG1-TFIG1hr40-rv	aatatccgctcaaaacttatatgtttatttgaggataaaattacttcgaagcctgacttggcagc
45	SacII-FIG1end50-ZsGreen-fw	aaaaccgcggataggtacaataactactcttcggattcatctacattgcattccaaagttgctcagt caaagcacggtct
46	ZsGreen-TFIG1hr40-rv	aatatccgctcaaaacttatatgtttatttgaggataaaatcagggcaatgcagatccggatgca
47	SacII-FIG1end50-EGFP(Y)-fw	aaaaccgcggataggtacaataactactcttcggattcatctacattgcattccaaagttgtcagta agggtgaagaattattcactggt
48	EGFP(Y)-TFIG1hr40-rv	aatatccgctcaaaacttatatgtttatttgaggataaaattacttgtataattcgtccataccc
49	SacII-FIG1end50-AcGFP1(Y)-fw	aaaaccgcggataggtacaataactactcttcggattcatctacattgcattccaaagttgtttctaa gggtgctgaatt
50	AcGFP1(Y)-TFIG1hr40-rv	aatatccgctcaaaacttatatgtttatttgaggataaaattacttgtataattcgtccatacca
51	SacII-FIG1end50-TagGFP2(Y)-fw	aaaaccgcggataggtacaataactactcttcggattcatctacattgcattccaaagtttctggtgg tgaagaattatt
52	TagGFP2(Y)-TFIG1hr40-rv	aatatccgctcaaaacttatatgtttatttgaggataaaatcatctgtataattcgtccataccg
53	SacII-FIG1end50-mUkG1(Y)-fw	aaaaccgcggataggtacaataactactcttcggattcatctacattgcattccaaagttgtcagtgt

		catcaaagaaga
54	mUkG1(Y)-TFIG1hr40-rv	aatatccgctcaaaacttatatgtttatttgaggataaaattacttagaagcttgagatggcaac
55	SacII-FIG1end50-ZsGreen(Y)-fw	aaaaccgcggataggtacaataactactcttcggattcatctacattgcattccaaagttgctcaat ccaaacatggttt
56	ZsGreen(Y)-TFIG1hr40-rv	aatatccgctcaaaacttatatgtttatttgaggataaaattatggcaaagcagaacctgaagca
57	FIG1end50-fw	ataggtacaataactactcttcggattcat
58	TFIG200-rv	aacagacggtaatgattagagtttaggtaa
59	Ste18pro-fw	atattatatatatatatagggtcgt
60	Ste18t-rv	aaattatagaaagcagtagataaaa
61	HIS3pro80-URA3-fw	tatataaagtaatgtgatttcttcgaagaatatactaaaaaatgagcaggcaagataaacgaagg caaagttcaattcatcattttttttttattctttt
62	HIS3t end40-rv	ggagccataatgacagcagttgggtaggcctttctttggt
